# Digital microfluidic isolation of single cells for -Omics

**DOI:** 10.1038/s41467-020-19394-5

**Published:** 2020-11-11

**Authors:** Julian Lamanna, Erica Y. Scott, Harrison S. Edwards, M. Dean Chamberlain, Michael D. M. Dryden, Jiaxi Peng, Barbara Mair, Adam Lee, Calvin Chan, Alexandros A. Sklavounos, Austin Heffernan, Farhana Abbas, Charis Lam, Maxwell E. Olson, Jason Moffat, Aaron R. Wheeler

**Affiliations:** 1 Department of Chemistry, University of Toronto, 80 St. George Street, Toronto, ON M5S 3H6 Canada; 2 Donnelly Centre for Cellular and Biomolecular Research, University of Toronto, 160 College Street, Toronto, ON M5S 3E1 Canada; 3 Institute of Biomaterials and Biomedical Engineering, University of Toronto, 164 College Street, Toronto, ON M5S 3G9 Canada; 4 Department of Molecular Genetics, University of Toronto, Toronto, ON M5S 3H6 Canada

**Keywords:** Lab-on-a-chip, Transcriptomics, Microfluidics, Proteomics, Biomedical engineering

## Abstract

We introduce Digital microfluidic Isolation of Single Cells for -Omics (DISCO), a platform that allows users to select particular cells of interest from a limited initial sample size and connects single-cell sequencing data to their immunofluorescence-based phenotypes. Specifically, DISCO combines digital microfluidics, laser cell lysis, and artificial intelligence-driven image processing to collect the contents of single cells from heterogeneous populations, followed by analysis of single-cell genomes and transcriptomes by next-generation sequencing, and proteomes by nanoflow liquid chromatography and tandem mass spectrometry. The results described herein confirm the utility of DISCO for sequencing at levels that are equivalent to or enhanced relative to the state of the art, capable of identifying features at the level of single nucleotide variations. The unique levels of selectivity, context, and accountability of DISCO suggest potential utility for deep analysis of any rare cell population with contextual dependencies.

## Abstract (web summary)

Multi-Omic approaches are a powerful way for obtaining in-depth understanding of a cell’s state. Here the authors present DISCO, combining digital microfluidics, laser cell lysis, and artificial intelligence-driven image processing to analyze single-cell genomes, transcriptomes and proteomes in a mixed population.

## Introduction

Single-cell -Omics analysis methods are having a transformative effect on research in the life sciences. Specifically, the capacity to assess genomes, transcriptomes, or proteomes of individual cells in place of (or in addition to) measuring the average states of populations of cells is leading to important advances in cancer biology^1,2^, neuroscience^3,4^, neural stem cell therapeutics^5,6^, and beyond. The microfluidics-based techniques that are driving this revolution (typically) rely on the partitioning of cells into droplets^7–11^, microchannels^12–15^, or microwells^16–21^, after which they are analyzed by the -Omics technique of choice. A drawback of these methods, however, is a lack of capacity to correlate the single-cell genomes, transcriptomes, or proteomes with the phenotypes of adherent cells in situ (e.g., size, shape, intracellular marker expression, distance to neighboring cells).

There are a handful of recently reported techniques that come close to bridging the divide between the analyses of single-cell -Omics and adherent phenotypes in situ. For example, Brasko et al.^22^ used laser capture microdissection (LCM) with machine learning to isolate the contents of single adherent cells and subsequently analyze housekeeping genes by PCR; however, the contents of tens-to-hundreds of cells were pooled together for each reported whole-transcriptome sequencing analysis. Likewise, Kamal et al.^23^ described a mechanical cell-picker technique that allowed for single-cell transcriptome sequencing, but reported a tendency to collect more than one cell per pick. Finally, Parker et al.^24^ described a creative method relying on photoelectrochemical effects to isolate individual cells from adherent culture prior to sequencing, but the technique requires specialized gridded substrates that force artificial limits on cell spacing. These techniques (and LCM, and mechanical pickers and patterned/specialty substrates, in general) are important and useful, but we are not aware of a robust, all-purpose path for the user to connect adherent cell phenotype to single-cell genomes, transcriptomes, or proteomes.

Here we introduce Digital microfluidic Isolation of Single Cells for -Omics (DISCO), a method that overcomes the limitations described above. In DISCO, a digital microfluidic (DMF) technique that facilitates automated culture, fixation, staining, and image-based analysis of adherent cells in situ^25^ has been paired with a custom artificial intelligence-based selection algorithm and laser-cell lysis (LCL), a technique originally developed as means to ablate cell contents into capillaries for electrophoretic analysis^26^. Herein we describe how DISCO is useful for capturing multi-Omics and image-based data from single cells selected from diverse, heterogeneous adherent cell populations. We have benchmarked the sensitivity and specificity of DISCO for genomics, transcriptomics, and proteomics analyses using human glioblastoma (U87) and murine melanoma (B16) cell lines, with additional proof-of-concept results identifying single-nucleotide-level differences in CRISPR-modified cells. We propose that DISCO represents an important tool in the single-cell analyst’s toolbox, that should be uniquely well suited for assessing questions related to the fundamental dogma of molecular biology, testing the relationship between genome, transcriptome, and proteome and phenotype.

## Results

### Digital microfluidic isolation of single cells for -Omics

DISCO is the first method to mate digital microfluidics (DMF)^27^ with laser cell lysis (LCL)^26^, a technique in which a focused, high-energy laser is used to lyse cells within a few micrometers of the focal point. LCL has been paired previously with conventional microfluidics, but bubbles were found to be problematic in the enclosed environment^28^. Bubbles are not a problem in the DMF format, as they simply diffuse away to the droplet-air interface.

**Fig. 1:**
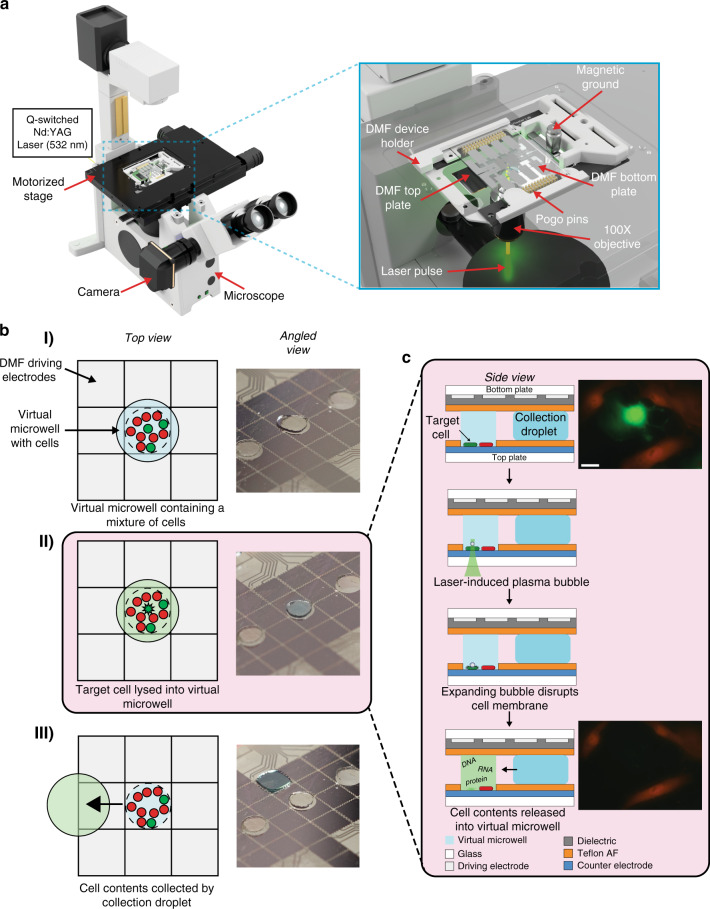
Digital microfluidic isolation of single cells for -Omics (DISCO). **a** Illustration of the platform used for DISCO (left). Zoom-in shows the integration of a DMF device into the microscope stage (right). **b** Top-view schematics (left) and angled-view photos (right) of a digital microfluidic device at various stages of processing. (I) Adherent cells (red and green) are cultured on a digital microfluidic device, and a collection droplet (blue) is positioned over the array of cells. (II) A single green cell is targeted for laser lysis into the collection droplet. (III) The collection droplet (green) is queued for -Omics analysis. **c** Side-view schematic (left) showing two adherent cells (red and green) cultured on a digital microfluidic device (top panel), and laser-induced plasma bubble formation and expansion (middle panels) causing cell lysis to release cell contents into droplet (bottom panel). Fluorescence microscopy images (right) of a co-culture of live eGFP-expressing U87 cells (green) and tdTomato-expressing B16 cells (red) on the DISCO platform before (top) and after (bottom) lysis of the U87 cell in the center. The scale bar is 50 μm.

The DISCO instrument comprises multiple components (Fig. 1a): a device for cell and reagent manipulation, an open-source Dropbot control system^29^ for droplet management on the DMF device, a microscope for imaging and selecting cells on the DMF device, and a Q-switched laser for cell lysis. The DMF device comprises two plates; the bottom plate contains a checkerboard array of driving electrodes that are used to manipulate droplets that can contain cells, reagents, or cell lysate, and the top plate contains sites for culturing adherent cells (formed here by a new sacrificial contact-mask method). Cell and reagent delivery and collection are facilitated by passive dispensing^30^, in which droplets spontaneously form “virtual microwells” on the basis of surface energy differences. The number of cells seeded into each virtual microwell is determined by the loading density; in experiments reported here, each microwell contained ~100–300 cells. Once loaded, each virtual microwell can be separately addressed with new droplets containing media or other reagents, which displace the original contents, allowing for fully automated immunocytochemistry^25^.

The process of cell selection, lysis, and collection into droplets by DISCO is shown in Fig. 1b. A key feature of the system is selectivity – the user can choose any cell or combination of cells of interest based on manual inputs to the custom control software (Supplementary Fig. S1), or alternatively the user can allow a custom artificial intelligence (AI)-driven selection process to automate the process. Upon selection of the cell(s) of interest, high-energy laser pulse(s) are delivered to the targeted cell(s). Each pulse generates a short-lived, highly localized plasma, which induces the formation of a cavitation bubble that expands and then collapses, disrupting the cell’s membrane and releasing its contents into solution^26^. This mechanism of lysis allows for rapid and efficient release of total cell contents, allowing for whole genome, transcriptome, or proteome collection, but limits the ability to collect contents from individual cellular compartments. The instrument described here features a focused laser with a spot size of ~0.8 μm, which provides precision sufficient to lyse a selected cell that is close to or attached to neighboring cells without disturbing them (Fig. 1c, Supplementary Movie 1), and the data presented here were collected from cells cultured on devices with a wide range of confluencies.

**Fig. 2:**
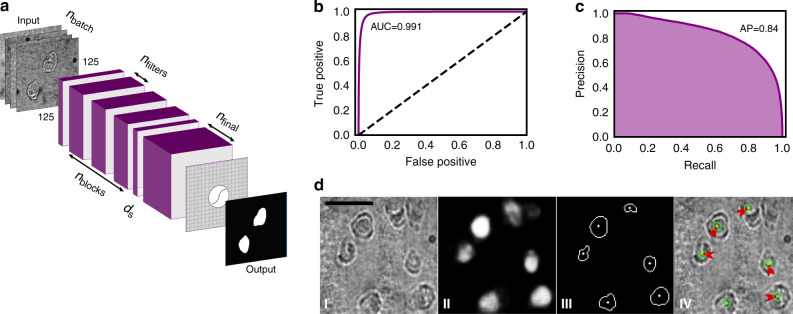
Artificial Intelligence for cell identification and lysis in the DISCO system. **a** Illustration of the architecture of the convolutional neural network (CNN) used for cell localization and segmentation with the image-inputs, prediction outputs, as well as the parameters used for hyper-optimization. The latter include the number of samples in each training batch (*n*_batch_), the number of intermediate blocks (*n*_blocks_), the number of filters in each of the intermediate blocks (*n*_filters_), the number of filters in the final layer (*n*_final_), and the dropout strength used between the intermediate layers and the final layer (*d*_s_). Each intermediate block contains 3 convolutional layers, and the images used as input to the network were resized to 125 × 125 px. **b** Receiver-Operator Characteristic (ROC) curve (purple) for the final hyper-optimized and trained network with area under the curve AUC = 0.991. The dashed line represents a ROC curve for pixel-wise random (i.e., coin-flip) guessing. **c** Precision-Recall curve for the network with average precision, AP = 0.84. **d** Representative AI predictions, post-processing, and machine vector instruction generation: (I) brightfield image captured at ×100 magnification and resized and intensity-normalized for input to the network, (II) network output cell position probabilities as pixel intensity values, (III) final cell edge and center positions after 50% probability thresholding and contour center calculation, and (IV) machine vector instructions (red arrows) and laser pulse emissions (green dot and circle) to be automatically executed by the LCL system for lysis of all cells in view. The scale bar is 100 μm.

Traditional approaches for microfluidic isolation of single cells have been limited by user-bias during cell identification and isolation^31^, which has led to the development of automated, artificial intelligence (AI)-driven techniques^22,32,33^ for cell identification. Inspired by this trend, we developed a custom convolutional neural network (CNN), which was trained (Supplementary Figs. S2–S4) and then used to segment images of cells in DISCO devices to identify their locations (independent of phenotype) for subsequent lysis (Fig. 2a). The optimized model had area-under-curve of 0.991 (Fig. 2b) and average precision of 0.84 (Fig. 2c), allowing for hands-free operation (Fig. 2d). In AI-driven experiments, the system is able to generate ~5 droplets containing the contents of a single cell per minute (Supplementary Movie 2), a rate that would scale to hundreds of cell contents collected per hour in an appropriately sized device. Modest improvements to throughput may be possible for future generations of DISCO (perhaps up to one thousand cell contents collected per hour), but we acknowledge that DISCO was not designed for throughput. DISCO is uniquely well suited to (i) select only cells of interest for sequencing from diverse populations of adherent cells, (ii) connect single-cell sequences to image-based phenotypes, and (iii) allow sequencing analysis by genomics, transcriptomics, and/or proteomics.

### Single cell genome analysis

In initial experiments, the well-characterized human glioblastoma U87 cell line^34^ was used to evaluate the performance of DISCO for single-cell genome sequencing. A custom single-cell genomics analysis pipeline (Supplementary Fig. S5) was developed and applied to ensure high data quality and efficient spending on sequencing reagents. For example, quality control test 1 (QC-1) tests for the presence of highly conserved loci on chromosomes 4, 12, 13, and 22 (Supplementary Fig. S6a) and QC-2 evaluates short tandem repeats (STRs) and other loci across 15 chromosomes (Supplementary Fig. S6b) using custom primers (Supplementary Table S1). After method optimization, approximately 95% of samples collected passed QC 1-2, and all samples that passed QC 1–2 resulted in high-quality sequences according to the pipeline’s bioinformatics tolerances (Supplementary Fig. S7).

**Fig. 3:**
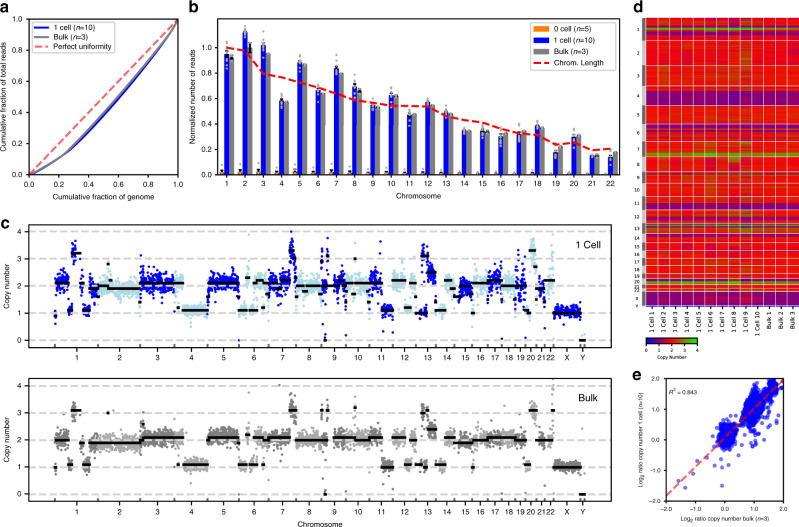
Single cell genomic analysis using DISCO. U87 cell monocultures were loaded onto DISCO devices, where they were fixed, targeted (0 or 1 cells per droplet), lysed, amplified, and analyzed according to the genome sequencing pipeline (Supplementary Fig. S5). For comparison, bulk samples of monocultures (~1000 cells) were also evaluated (foregoing the pipeline, with no amplification). **a** Lorenz curve of the average cumulative fraction of mapped reads as a function of the cumulative fraction of the U87 genome for single cells (“1 Cell,” blue, *n* = 10), and bulk samples (“Bulk,” gray, *n* = 3). The dashed red line represents a theoretical perfect match between reads and genome. **b** Plot of normalized number of reads as a function of chromosome for DNA collected from DISCO experiments with (blue, *n* = 10) and without (orange, *n* = 5) lysis of a single cell, and bulk samples (gray, *n* = 3). Error bars represent ±1 SEM, gray dots represent individual replicates, and the dashed red lines illustrate the length of each chromosome and the theoretical read distribution across the genome. **c** Representative plot of copy numbers measured for DNA extracted a single cell (top) and from bulk DNA (bottom). Data are plotted as a function of chromosome number, with dark blue, lt. blue, dark gray, and lt. gray circles representing the estimated copy numbers as determined by read depth analysis of bins containing 500 kb of uniquely mappable sequences and black line-segments representing the segmented medians as determined by circular binary segmentation. **d** Heatmap (with green-high and blue-low) of DNA copy numbers measured from single cells (*n* = 10) and bulk samples (*n* = 3). Each column represents a replicate, which are plotted as a function of location in each chromosome (rows). The color bar (bottom) illustrates the estimated DNA copy number. **e** Log_2_ correlation plot of single-cell and bulk data from **d**. Each circle represents the estimated copy number. A linear least squares regression fit (dashed red line) and coefficient of determination (*R*^2^ = 0.843) are shown.

In initial DISCO/genomics experiments, Lorenz curves were generated from the genomes of single cells and from bulk samples comprising pooled lysates of ~1000 cells, to assess amplification biases in single cell genomes potentially arising from multiple displacement amplification (MDA) (Fig. 3a). The results suggest that there is minimal bias introduced by MDA, and the near-theoretical distribution of reads across the genome demonstrates high sequencing uniformity that compares favorably to single-cell genomes obtained recently by limiting dilution^35,36^. Next, the average numbers of reads per chromosome were evaluated for single cells and bulk samples, as well as from negative-control “0 cell” samples, which were generated from droplets incubated with cells on devices but without lysis (Fig. 3b). The latter were tested to assess cross-talk and cross-contamination from cells that were not selected for lysis (which has been reported for LCM^37^ and mechanical cell picking^23,31^). The negative controls confirmed negligible background DNA (<5% compared to bulk) across all chromosomes, with read coverage from single cells comparable to those of bulk controls, both of which correlate with the theoretical read distribution across the genome. The only exception is for chromosome 4, which shows a decreased number of reads, which is unsurprising given that human glioma cell lines are known^38^ to have copy number losses in this chromosome. These initial characterizations demonstrated genome coverage uniformity, minimal introduction of amplification bias and little genomic contamination from non-selected cells in single-cell genomes generated using DISCO.

Given that cancer cells are known to have copy number variants (CNVs), we performed a genome-wide CNV analysis on single-cell and bulk samples. For most single cells analyzed, CNVs were similar to those observed for bulk samples (Fig. 3c), which is unsurprising given the homogeneity in cell lines. However, in considering CNVs within each single cell individually, it was found that there are subtle differences in copy number between cells. For example, in Fig. 3d, cell 8 has 3 copies of a portion of chromosome 8 instead of 2 copies, and 2 copies of a portion of chromosome 20 instead of 1 copy, highlighting the sensitivity of DISCO to assess CNVs between single cells. Finally, we compared the average copy numbers of each sample (single-cell and bulk) (Fig. 3e), revealing close agreement between the two, with *R*^2^ = 0.843. The same analysis was applied to compare the genomes of single cells selected manually and by AI (Supplementary Fig. S8). As expected, these groups also had a strong correlation (*R*^2^ = 0.894), giving us confidence that the AI system does not introduce unwanted bias into the pipeline. In sum, the CNV analysis provides a broad view of the functional output for DISCO as applied to single-cell genomics, illustrating the sensitivity to evaluate CNVs between single cells.

Lastly, to evaluate the capacity to select single cells in a heterogeneous environment, a mixture of human-derived U87 cells (transfected with eGFP) and mouse-derived B16 cells (transfected with tdTomato) were seeded and cultured on DMF devices (as in Fig. 1c), followed by selection and collection of single-cell lysates. Each set of samples was carried through the pipeline (Supplementary Fig. S5), with slight modifications to the QC steps (representative data in Supplementary Fig. S9a). Samples were sequenced and the mapping-rate efficiencies for mouse and human genomes were determined for single-cell and bulk analyses (Supplementary Fig. S9b), showing mapping rates >80% to the correct species and <8% to the incorrect species. These data confirm the high selectivity of DISCO for targeted cells in a heterogeneous population, and strengthen our confidence that genetic material from non-selected cells does not confound the results.

### Single cell transcriptome analysis

A DISCO pipeline was developed for transcriptome sequencing in single cells (Supplementary Fig. S10) featuring a custom poly-A tail-based extraction and custom, barcoded amplification (Supplementary Table S2), and bioinformatic analyses for cell demultiplexing (Supplementary Fig. S7). Initial characterization involved determining effects of cell-number on transcriptome coverage in B16 cells, evaluating droplets containing the lysates of 0, 1, or 2 cells generated by DISCO, in comparison to bulk samples comprising pooled lysates of ~1000 cells (prepared by chemical lysis). As expected, as more cell contents are combined, greater numbers of genes are detected, with fewer transcripts devoted to each of them (Supplementary Fig. S11a). But for robustly expressed genes, samples showed similar expression patterns regardless of the number of cells captured as input or lysis mechanism (Supplementary Fig. S11b). In addition, as is the case for genomic analysis (Supplementary Fig. S8), little discrepancy was found between transcriptomes generated in AI versus manually selected single cells (Supplementary Fig. S12). Finally, a standard-spike-in analysis (Supplementary Fig. S13) suggests that technical noise contributions to the analysis are predictable, which may make normalization to isolate biological variations feasible in the future.

**Fig. 4:**
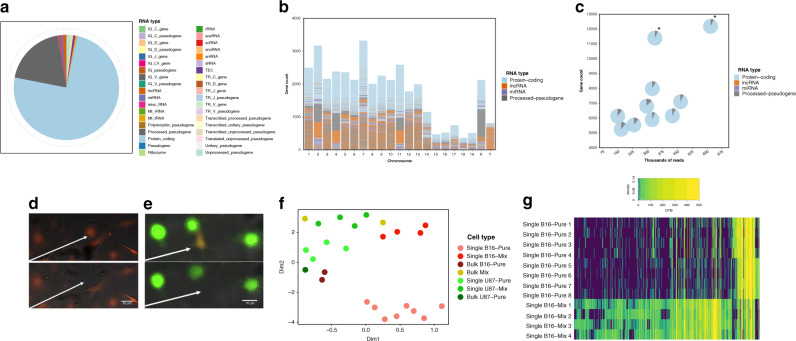
Single cell transcriptomic analysis using DISCO. B16 cell or U87 monocultures or U87/B16 co-cultures were loaded onto DISCO devices, where live cells were targeted (1 cell per droplet), lysed, and analyzed according to the transcriptome sequencing pipeline (Supplementary Fig. S10). For comparison, bulk samples of monocultures or co-cultures (~1000 cells) were also evaluated (foregoing the pipeline, analyzed directly by sequencing). **a** Pie chart showing consolidated gene expression from *n* = 8 representative single B16 cells binned into the type of RNA the expression originated from **b**. Plot of the protein-coding RNA (blue), lncRNA (orange), miRNA (purple) and processed pseudogenes (gray) as a function of chromosome for the data-set from **a**. **c** Plot of the number of genes detected per cell as a function of aligned reads for the data-set from **a**, compared to two bulk samples (labeled with asterisks). Each marker is a pie-chart broken into the categories from **b**. Fluorescence microscopy images of live cells on DISCO devices collected before (top) and after (bottom) laser lysis from (**d**) a B16 cell monoculture or (**e**) a co-culture of B16 (red) and U87 (green) cells. White arrows indicate the position of the particular B16 cell selected for lysis. **f** Plot of UMAP reduction to two dimensions (Dim1 and Dim2) of transcriptomes generated from (i) single B16 cells from monoculture (pink, *n* = 8) or co-culture (med. red, *n* = 4), (ii) single U87 cells from monoculture (med. green, *n* = 4) or co-culture (lt. green, *n* = 4), and (iii) bulk cells from B16 monoculture (dark red, *n* = 2), co-culture (yellow, *n* = 2), or U87 monoculture (dark green, *n* = 1). **g** Heat map of gene expression level in counts per million (CPM, with purple-low, yellow-high) of 5300 differentially expressed transcripts (determined by the quasi-likelihood F test, EdgeR) for single B16 cells from monoculture (top 8 rows) and from co-culture (bottom 4 rows); the corresponding list and expression values are found in Supplementary Data 1. The white trace labeled “density” in the legend represents the distribution of the different normalized expression levels in the data-set.

The composition of RNA detected from individual B16 cells was then evaluated to determine the types of genes that were expressed (Fig. 4a) and their locations in the genome (Fig. 4b). As expected for a technique relying on poly-A tail-extraction, the majority of sequences detected are from protein-coding mRNAs; however, there are also substantial signals detected from other kinds of RNA (including lncRNA and miRNA), and more surprisingly, there are a number of processed pseudogenes. Single cell transcriptomes had a similar compositional breakdown of gene-types (Fig. 4c), with comparable results observed for single U87 cells (Supplementary Fig. S14). In sum, these analyses highlight the diversity of gene types detected in the transcriptomes of single cells, while also showcasing the consistency in captured RNA content between them.

As indicated above, a unique strength of DISCO is the capacity to select particular cells for analysis on the basis of environmental cues in heterogeneous populations. To test this capacity, transcriptomes were generated from single B16 cells (murine) found in monocultures (Fig. 4d) and in B16 cells surrounded by U87 cells (human) (Fig. 4e). This kind of analysis can lend insight into the often competitive relationship exhibited by transplanted human cells in resident murine cells in the brain^39,40^. The transcriptome profiles were found to be radically different for B16 cells collected from monoculture versus co-culture. As shown in Fig. 4f, this was apparent even after dimension reduction of the global transcriptomic signature to a single point in 2D space. The differences are also evident at the transcript level – for example, 5300 transcripts were found to be differentially expressed (with a false discovery rate, FDR < 0.05, using the quasi-likelihood F test provided by EdgeR), in comparing B16 cells selected from monoculture versus a co-culture where they were surrounded by U87 cells (Fig. 4g, annotations and corresponding expression values are found in Supplementary Data 1). Upon evaluating this list with EnrichR, pathways involved in filopodia tip generation (GO:0032433, adjusted *p* = 0.03) and phosphatidylinositol signaling (KEGG mouse 2019, adjusted *p* = 0.004) were found to be enriched in cells selected from monocultures, whereas pathways such as electron transport chain (Wiki mouse pathways, 2019, adjusted *p* < 0.001) and oxidative phosphorylation (Wiki mouse pathways, 2019, adjusted *p* < 0.001) were found to be enriched in cells selected from co-cultures, signifying a difference in B16 cell behavior contingent upon presence of surrounding U87 cells. In sum, the selectivity permitted by DISCO allows the contribution of contextual metadata to further probe how cell-to-cell interactions affect transcriptional signatures of individual cells.

### Single cell proteome analysis

**Fig. 5:**
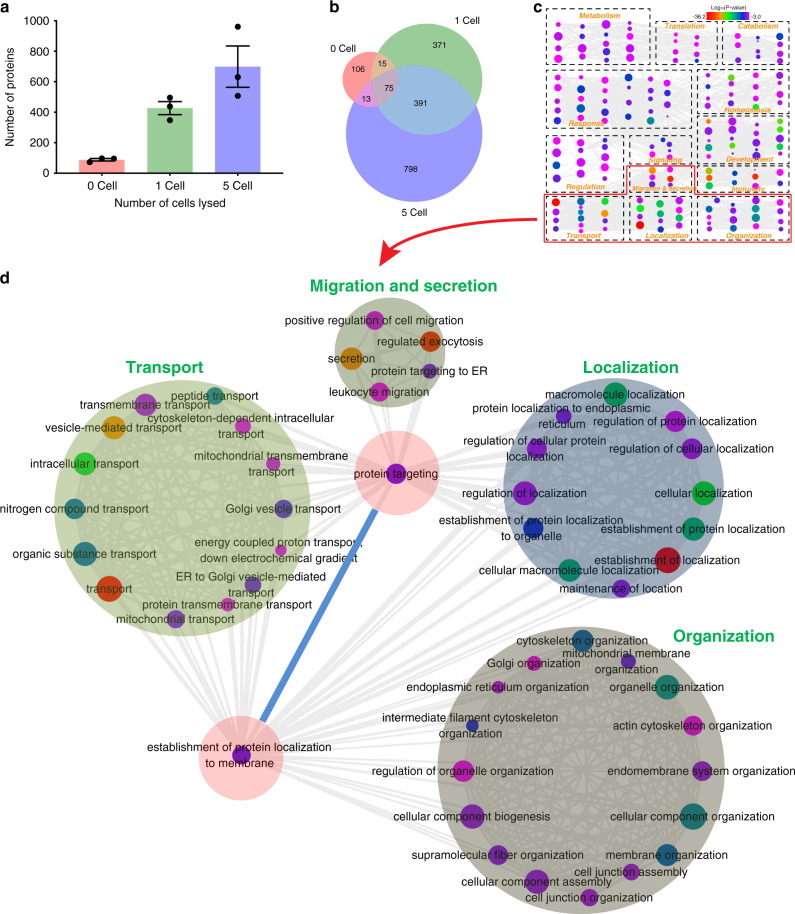
Single cell proteomic analysis using DISCO. U87 cell monocultures were loaded onto DISCO devices, where they were fixed, targeted, lysed, digested, and analyzed according to the proteome sequencing pipeline (Supplementary Fig. S15). **a** Plot of the number of proteins detected from U87 cell lysate droplets containing 0, 1, and 5 laser lysed cells. Black circles represent individual replicates and error bars represent ±1 SEM for three replicates per condition. **b** Venn diagram of overlapping protein identities from the data in **a**. **c** Graphical representation of biological process for the proteins identified in single U87 cells (*n* = 3) using DISCO. Dashed boxes are groupings according to biological process, marker color represents the Log_10_(*p*-value) corresponding to statistical confidence of protein function, marker size represents the frequency of the gene-ontology (GO) term in the underlying gene-ontology annotation (GOA) database, and gray lines indicate the correlation between biological functions. **d** Graphical representation of detailed relationships between proteins identified in single U87 cells with functions related to transport, migration and secretion, localization, and organization.

Recently developed technologies allow users to analyze the genomes, transcriptomes, proteomes, and various combinations of two of the above^10,35,41^, but to our knowledge there are no previous reports of one platform used for all three. A custom single cell proteomic pipeline (Supplementary Fig. S15) was developed to collect cell lysates generated by DISCO, evaluate them by LC-MS/MS, and identify the set of proteins detected after custom bioinformatics tolerances were applied (Supplementary Fig. S7). We first assessed the number of proteins identified in U87 cells (Supplementary Data 2, Fig. 5a). As shown, the numbers of proteins identified in each sample (mean ± SEM) were 88 ± 8, 427 ± 43, and 699 ± 136 for 0, 1, and 5 cells, respectively, numbers that are comparable to those reported recently in single-cell proteomics studies^42^. An analysis of the types of proteins identified (Fig. 5b) shows a strong overlap between the 1-cell and the 5-cell samples and minimal overlap with the 0-cell sample, which (like the data in Fig. 3b and Supplementary Fig. S11a) supports the conclusion that the samples are not substantially contaminated with proteins from non-selected cells. The proteins identified within single-cell samples were categorized according to their functions (Fig. 5c), revealing that many of the hits correlated to organization, transport, localization, migration and secretion. In particular, significant amounts were found for protein transport protein Sec61 subunit gamma (SEC61G) and beta (SEC61B), and ubiquitin-40S ribosomal protein S27a (RPS27A), which are all involved in membrane protein translocation^43^ and targeting of cellular proteins for degradation (Fig. 5d)^44^. These proteins have also been shown to be over expressed in glioblastoma patients^45^ and glioblastoma stem cells^46^, validating the results achieved here. These results demonstrate the capacity to identify proteins involved in a wide range of cellular pathways, with selectivity mirroring the genomics and transcriptomics results, and further stretching the utility of DISCO for the collection of intact proteomes from single cells.

### Genotyping of CRISPR modified HAP1 cells using DISCO

**Fig. 6:**
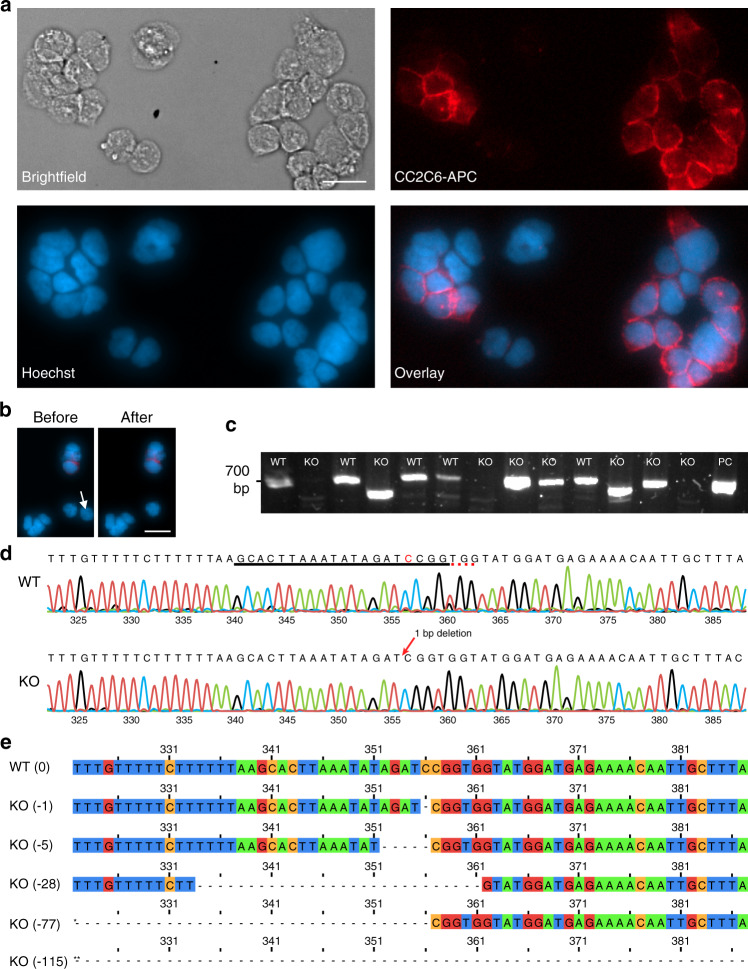
Monitoring variations in CRISPR modified cells using DISCO. CRISPR modified HAP1 cells were loaded onto DISCO devices, where they were fixed, stained, imaged, targeted (1 cell per droplet), lysed, the gRNA target locus was PCR-amplified from the genomic DNA, and analyzed by Sanger sequencing. **a** Microscopy images [left-to-right from top-left–Brightfield; anti-CD47 antibody (red); Hoechst for nuclear staining (blue); Overlay (blue and red)] of immunofluorescently stained cells on a DISCO device. Each cell was identified as WT (Hoechst^+^ CD47^+^) or KO (Hoechst^+^ CD47^−^) on the basis of staining. **b** Fluorescence microscopy images of cells stained identically to (A) before (left) and after (right) lysis of a single KO cell (white arrow). The scale bars for (**a**–**b**) are 20 μm. **c** Image of an agarose gel after electrophoretic separation of PCR products from targeted amplification of CD47 for DNA samples originating from single WT (*n* = 5) and KO (*n* = 8) cells selected by DISCO, as well as positive control (PC). **d** Representative electropherograms (base-numbers on *x*-axes) from Sanger sequencing analysis of a single WT (top) and a single KO (bottom) cell. The CD47 sgRNA sequence is underlined in black and the PAM sequence is underlined in the red dotted line. The cytosine-deletion causing the KO is indicated in red font. Colored peaks in the electropherogram correspond to the nucleotide call (green = A, black = G, red = T, blue = C). **e** Representative nucleotide sequences from the CRISPR target region (340-359 base pairs) for one WT cell (top) and five KO cells (bottom). The KO cells have base-pair deletions of 1, 5, 28, 77*, and 115** in the sequence-region shown. The latter two extend to 280* and 273** base pairs so the full deletion is not shown.

After confirming the performance of DISCO for robust single-cell -Omics measurements, we applied it to an important application – evaluating the correlation between phenotype and genotype after genetic modification by CRISPR. We used CRISPR-modified HAP1 cells, which are commonly used for loss of function genetic screens^47,48^, transduced as reported previously^49^ with Cas9 and a single-guide RNA (sgRNA) targeting CD47, a cell surface protein responsible for immune evasion in cancer. DISCO was used to culture, fix, label for immunofluorescence, and image the cells. As expected, there was heterogeneity in the phenotypes of the modified cells (Fig. 6a), with some cells exhibiting normal expression of CD47, which we refer to as wild-type (WT) cells, while others exhibited low or no CD47 expression, which we refer to as knockout (KO) cells. DISCO was used to select and lyse single WT and KO cells, as illustrated in representative images before and after laser lysis in Fig. 6b. Single-cell contents were propagated through the genomics pipeline (Supplementary Fig. S5), with PCR and Sanger sequencing approaches as an alternative to NGS to test for mutations in the CD47 gene that cause a frameshift and/or deletion and lead to truncation of the resulting protein or degradation of the mRNA. PCR primers were designed to amplify a ~700 bp fragment around the target locus on the genomic DNA, and the results (Fig. 6c) show the expected ~700 bp product for WT cells (lanes 1, 3, 5, 6, and 10) and three different types of fragments from KO cells: (1) similar size to WT (lanes 8, 9, and 12), (2) noticeably smaller (lanes 4 and 11), and (3) absence (lanes 2, 7, and 13).

The results for samples which produced bands on the gel were confirmed by Sanger sequencing, with representative electropherograms for a typical WT and a representative KO cell (featuring a single-nucleotide deletion) shown in Fig. 6d. The sequences were aligned (Fig. 6e), and of the eight KO cells (from Fig. 6c) analyzed, four had large deletions (three with deletions of almost the entire ~700 bp PCR product, and another with a 115 base pair deletion), two had medium-size deletions (28 and 77 base pair deletions) and two had small deletions (1 and 5 base pair deletions) (Supplementary Table S3). The capability to probe selected cells’ sequences at high resolution is unique to DISCO; for many of the high-throughput microfluidic techniques, the operator must set sequencing parameters for the entire pool of single cells (rather than for particular cells of interest), which is often prohibitively expensive. Finally, immunofluorescent labeling intensity for CD47 (from image analysis) was compared to small, medium, and large deletions, showing a significant (*p* < 0.05) decrease in CD47 expression for WT versus all deletions (Supplementary Fig. S16). In sum, this application of DISCO highlights three unique features of the technique: DISCO has the flexibility to be coupled with diverse -Omics techniques (e.g., genotyping by NGS as in Fig. 3 and Sanger sequencing as in Fig. 6), DISCO allows for sensitive analyses capable of detecting single nucleotide mutations (as well as larger genomic variations between individual cells), and DISCO is capable of associating genomic composition with phenotypic assessments.

## Discussion

DISCO (Figs. 1, 2) is an addition to the canon of microfluidic techniques that have been developed for single-cell -Omics analysis^7–21^. A disadvantage of DISCO relative to some in this list is throughput; techniques like Drop-Seq^7^ and the 10× Genomics platforms^9^ can evaluate the transcriptomes of many more cells per unit time. But in contrast to existing systems, DISCO provides the user with the capacity to (i) select only cells of interest for sequencing from a small initial sample, (ii) connect single-cell sequences to image-based phenotypes, and (iii) allow sequencing analysis by multiple -Omics techniques without the requirement of physically separating or dissociating single cells from their neighbors. We are not aware of any previous technique with this unique set of capabilities.

DISCO was designed to enable the collection of single-cell genomics, transcriptomics, and proteomics data at levels that are equivalent to the state of the art. Specifically, the genomics analyses demonstrated here show high reproducibility between replicates, sensitivity in detection of single-cell CNV heterogeneity, and highly specific single cell isolation (Fig. 3). The transcriptomics analyses feature robust and reproducible detection of a diversity of RNA, as well as the sensitivity and selectivity to detect transcriptional shifts resulting from surrounding cell composition (Fig. 4). And rounding out the assessment of the central dogma of molecular biology, the proteomics analyses facilitated by DISCO identified numbers of proteins per single cell that are comparable to most advanced label-free single-cell proteome analysis systems that have been reported previously^42^ (Fig. 5), while exhibiting detection of proteins from a diversity of cellular compartments, as well as pathways. Lastly, functionality and scalability of DISCO was exhibited by the identification of single nucleotide variations (SNVs) between individual cells modified by the CRISPR-Cas9 system, resulting in information that can (uniquely) be correlated with image-based phenotypes (Fig. 6).

The levels of context and accountability of the single cell analyses produced by DISCO provide some of the most efficient usage of single cell -Omics data to date, with the potential to extend its utility to any rare cell population, while incorporating contextual dependencies. Recently, there have been several exciting reports^50–52^ of methods that can capture transcriptomic signatures of cells residing in histological grade tissue slices (preserving spatial information about the samples); however, these techniques have not (yet) been demonstrated for single-cell resolution, and they are by nature limited to transcriptomics. The application of evaluating tissue slices is an important one, and although not addressed here, we propose that application represents an exciting horizon for DISCO studies in the future.

## Methods

### Reagents and materials

Unless otherwise specified, reagents were purchased from Sigma Chemical. Deionized (DI) water had a resistivity of 18 MΩ•cm at 25 °C. 10% Pluronic F68 solution, Iscove’s Modified Dulbecco Medium (IMDM), Dulbecco’s modified Eagle medium (DMEM), Dulbecco’s Phosphate-Buffered Saline (DPBS) with no calcium and magnesium, and fetal bovine serum (FBS) were purchased from Life Technologies. Teflon-AF 1600 was purchased from DuPont.

### DISCO device fabrication

DISCO devices, each comprising a bottom plate and top plate, were fabricated in the University of Toronto Nanofabrication Centre (TNFC) cleanroom facility, using transparent photomasks printed at 20,000 DPI (Pacific Arts and Designs Inc.). The bottom plates of DISCO devices bearing arrays of chromium electrodes coated with dielectric and hydrophobic layers were formed using methods similar to those described previously^25^. The design features an array of 80 roughly square actuation electrodes (2.2 × 2.2 mm ea.) interfaced with 8 rectangular reservoir electrodes. Within the array of actuation electrodes is a linear pattern of eight circular “windows” (1.75 mm dia., 4.4 mm pitch) that are free from chromium (to permit bright-field imaging). Each driving electrode is connected to a contact pad designed to interface with a custom pogo-pin connector on the instrument (details below). After processing, bottom plates were sterilized using 70% ethanol, air dried, and stored until use.

DISCO device top plates, bearing hydrophilic cell culture sites, were formed from 3ʺ × 1ʺ indium-tin oxide (ITO) coated glass substrates (Delta Technologies Ltd) by a sacrificial contact-mask method. Briefly, substrates were cleaned with acetone, isopropanol, DI water and drying with nitrogen gas, and the ITO layer was covered in dicing tape (Semiconductor Equipment Corp., Moorpark, CA). A 40-W H-Series Desktop CO_2_ Laser (Full Spectrum Laser) was used to generate a linear pattern of eight free-standing circular discs (1.75 mm dia., 4.4 mm pitch), which served as sacrificial contact masks. The excess dicing tape was removed, and the substrates were spin-coated with Teflon-AF 1600 (2% w/w in Fluorinert FC40, 2000 RPM, 30 s) and baked in an oven (165 °C, 10 min). The sacrificial contact masks were removed, and the substrate was cleaned with acetone, isopropanol and 70% ethanol, air dried, and stored until use. To assemble completed DISCO devices, a top plate was joined by a spacer formed from two pieces of Scotch double-sided tape (3 M) or double coated white polyester diagnostic tape 9965 (3 M) with total spacer thickness of ~180 µm. Care was taken during this process to align the cell culture sites on the top plate with the windows on the bottom plate. As described below, in some cases, assembled devices were used for an automated cell seeding and processing procedure, while in other cases, cells were seeded onto top plates manually prior to assembling with bottom plates.

### Cell culture

Cells were grown in a humidified incubator at 37 °C with 5% CO_2_. HAP1 cells were CRISPR-modified as reported previously^49^ and were grown in IMDM. U87 and B16 were stably transfected with eGFP and tdTomato^53^, respectively (kindly provided by Prof. Warren Chan, Univ. Toronto), and were grown in DMEM. All media contained 100 U/mL penicillin G and 100 µg/mL streptomycin and was supplemented with 10% FBS. Cells were passaged by trypsinizing, pelleting, washing in PBS, and resuspending in fresh media at desired densities (determined by hemocytometer). “Bulk” samples were formed by pelleting ~1000 cells (or more) and resuspending in appropriate media prior to analysis for genome, transcriptome, or proteome sequencing (details below).

### DISCO instrument and operation

The DISCO instrument is built on the body of an Olympus IX-71 inverted microscope with a motorized stage (Prior Scientific, Model H117P2IX) and a 14 MegaPixel, 30 frames-per-second camera (OptixCam, Summit SK2). A frequency doubled (532 nm) Q-switched Nd:YAG laser (Quantel, Ultra 50) is integrated with the rear lamp house port of the microscope, and is focused through a ×100 objective (BoliOptics, NA = 0.8) onto the inner surface of a DISCO top plate (where cells are adhered) for laser cell lysis. The stage, laser, and camera are controlled via a custom software system (Supplementary Fig. S1). Cells are selected manually or by a custom AI algorithm (described below). Lysis is achieved by firing one or more 8 ns pulses of the laser near the center of the selected cell at high power (4.5 μJ) when cells are far apart or around the edges of a selected cell at low power (1.5 μJ) when cells are close together.

The microscope stage features a custom insert to hold DISCO devices. The insert houses an array of pogo pins and other electrical connectors, serving as the interface between the DISCO device and the open-source DropBot^29^ control system. Droplet position is programmed and managed by application of a series of sine wave pulses [100–110 V_rms_, 10 kHz, conditions determined to be below the saturation forces^54^ for the liquids used here] between bottom plate electrodes and the top-plate counter electrode.

### Convolutional neural network development and optimization

For cell segmentation and localization, a convolutional neural network (CNN) based on a modified DenseNet architecture^55^ was employed for pixel-wise binary crossentropy classification. In total, 332 bright-field images were acquired using the DISCO system (above), annotated via custom software, and then used for training with image augmentation. The images contained pictures of both live and fixed U87 and B16 cells in approximately equal proportions and at differing stages of confluency. The images were annotated in a binary fashion (i.e., the initial model used here was not trained to differentiate between cell types—only to determine cell locations within the field of view) so training set class imbalance was not critical. Fig. 2a shows the architecture of the network, which consists of stacked blocks of convolutional layers followed by a dropout layer and then a final dilated convolutional layer with a larger number of filters. To improve performance, model architecture parameters were used as variables for hyperparameter optimization. Along with the learning rate and training batch size, the other hyperparameters optimized were the number of convolutional layers, the number of filters within each layer, and the dropout strength. Additional information regarding the model-generation is shown in Supplementary Figs. S2 and S3.

When deployed for laser lysis, the pixel-wise probabilities output by the model were post-processed using a binary threshold of 0.5, and the contours of the positive class were used for laser targeting. This manner of precise segmentation allowed for two modes of laser lysis: (1) “direct” targeting, and (2) “excision,” which can be selected by the user to be applied globally (for complete automation) or on a cell-by-cell basis (for semi-automated processing). Either way, the lysis process is triggered via the GUI (Supplementary Fig. S1): the user inputs the number of desired cells to lyse, clicks the “Process Well” button, and the stage spirals outward from the center of the well, during which time the camera input is fed into the CNN model. When a cell is selected, the system centers the laser spot in the centroid of the cell (for “direct” mode) to fire a single laser pulse, or the edge of the cell (for “excision” mode), after which the stage actuates around the contour of the cell as the laser is pulsed at its max repetition rate (20 Hz). Once the number of desired cells has been lysed into a given droplet, the system returns manual control to the user. [Currently, centering the camera within each well and adjusting the focus is performed by the user; this will be automated in future generations of DISCO.]

### Cell seeding, processing, and selection on DISCO devices

To initiate a DISCO experiment, cells were seeded and processed on DISCO devices: steps 1-2 in the pipelines (Supplementary Figs. S5, S10, S15). In some experiments, these steps were implemented in an automated procedure on devices with top and bottom plates that were loaded into the DISCO instrument. Each sub-step of the procedure comprised loading a reagent into a reservoir, dispensing eight 2-μL droplets across the cell culture sites to effect passive dispensing^30^ into virtual microwells (and driving the spent droplets to a waste reservoir), and incubating the device at given temperature/humidity and duration. (Note: when not room temperature, the device was temporarily removed from the DISCO instrument to a separate chamber, and then returned to the instrument after.) The reagents and incubation conditions for each sub-step are indicated in the following list, where cells destined for analysis by genomics (5 sub-steps), transcriptomics (2 sub-steps), or proteomics (5 sub-steps) are indicated (respectively) with suffixes -g, -t or -p. (sub-step i-g,t,p) reagent: cells suspended in media at 0.5 × 10^6^ cells/mL; incubation: 37 °C, humidified atmosphere, 24 h. (sub-step ii-t) reagent: DPBS with 0.05% (v/v) F68; incubation: none (at completion, ready for cell selection and lysis). (sub-step ii-g,p) reagent: HOPE® Fixative solution I (Polysciences, Warrington, PA); incubation: 4 °C, 20 min. (sub-step iii-g,p) reagent: DPBS with 0.05% (v/v) F68; incubation: none (wash). (sub-step iv-g,p) reagent: ice-cold acetone; incubation: −20 °C, 10 min. (sub-step v-g) reagent: DPBS with 0.05% (v/v) F68; incubation: none (at completion, ready for cell selection). (sub-step v-p) reagent: DPBS with 0.0125% (v/v) n-Dodecyl β-D-maltoside (DDM); incubation: none (at completion, ready for cell selection). In other experiments, cells were seeded and processed manually (using a pipette to dispense and aspirate in place of passive dispensing), replicating the conditions indicated above but on open top plates. In these experiments, after completion, top-plates were joined with bottom plates and loaded into the DISCO instrument for cell selection and lysis. (Note that devices bearing HAP1 cells were subjected to additional processing between steps 2 and 3, described in a section that follows.)

The next stage of a DISCO experiment is automated cell selection and lysis: steps 3-4 in the pipelines (Supplementary Figs. S5, S10, S15). Sub-step (I) comprised loading a reagent into a reservoir (DPBS with 0.05% v/v F68 for cells destined to be analyzed by genomics or transcriptomics, or DPBS with 0.0125% v/v DDM for cells destined to be analyzed by proteomics), dispensing eight 4-μL droplets across the cell culture sites to effect passive dispensing into virtual microwells (and driving the spent droplets to a waste reservoir). Sub-step (II) comprised selecting zero, one, or more cells to be lysed into their respective virtual microwells (process described above). Sub-step (III) comprised a repeat of sub-step (i), except the spent droplets were driven to a collection port for subsequent aspiration by pipette into a PCR tube. Samples generated in this manner were stored at −20 °C or −80 °C until processing for genomic, transcriptomic, or proteomic analysis. When interfaced with the AI algorithm, the durations of sub-steps (I), (II), and (III) were approximately 5.5 s, 1 s, and 5.5 s, yielding a total of around 12 s per cell. (Supplementary Movie 2 depicts sub-steps II–III.) In some experiments, sub-steps (I–III) were repeated iteratively to select different cells (sequentially) from the same virtual microwells.

### Genomic analysis of single cells

Aliquots of cell lysate (4 µL) collected from DISCO devices were subjected to step 5 of the genomics pipeline (Supplementary Fig. S5): whole genome amplification (WGA) using the Repli-g single cell kit (Qiagen) following the manufacturer’s instructions. The amplified product was vortexed vigorously for 10 s, heated at 65 °C for 10 min to shear the long stranded genomic DNA, and diluted 1:50 in ultrapure DI water.

Next, samples were subjected to step 6 of the genomics pipeline (Supplementary Fig. S5): quality control 1 (QC-1). In this step, particular loci in a 2 µL aliquot of 1:50 diluted WGA product were amplified by PCR using Taq DNA Polymerase (Invitrogen) according to the manufacturer’s instructions, and then were evaluated by gel electrophoresis on a 4% w/v agarose gel, visualized using a Gel Doc^TM^ EZ Imaging System (Bio-Rad). Samples derived from human U87 and HAP1 monocultures were tested for loci on chromosomes 4, 12, 13, and 22 as described previously^56–58^, using primers found in Supplementary Table S1. Typical data are shown in Supplementary Fig. S6a; samples with at least 3 of the 4 expected loci were carried forward through the pipeline (with others discarded). Samples derived from U87/B16 co-cultures were tested for GFP and tdTomato (primer sequences found in Supplementary Table S1). Typical data are shown in Supplementary Fig. S9a; samples with the expected loci (U87^−^GFP, B16-tdTomato) were carried forward through the pipeline (with others discarded).

Next, samples were subjected to step 7 of the genomics pipeline (Supplementary Fig. S5): DNA-cleanup. The 1:50 diluted MDA products were purified using DNA Clean and Concentrator PCR purification columns (Zymo Research) according to manufacturer’s instructions, with a final elution volume of 25 μL in ultrapure DI water. A Qubit ®4.0 Fluorometer (Invitrogen) was used to quantify the concentration of eluted DNA.

Next, samples were subjected to step 8 of the genomics pipeline (Supplementary Fig. S5): quality control 2 (QC-2), in which aliquots of purified DNA equivalent to 1 ng were amplified by PCR and evaluated by electrophoresis. Specifically, samples derived from U87 cells were amplified using methods similar to those described previously^59^ at fifteen short tandem repeat (STR) loci and the locus for Amelogenin using the AmpFLSTR Identifiler PCR Amplification Kit (Applied Biosystems) according to manufacturer’s instructions, followed by analysis by microfluidic gel electrophoresis on an Agilent 2100 Bioanalyzer. Typical results are shown in Supplementary Fig. S6b; samples derived from U87 cells with 12 of 16 loci were carried forward through the pipeline (with others discarded). Samples derived from mouse B16 cells were amplified and analyzed identically to those from U87 cells, but for loci on chromosomes 3, 6, and 15, using primers found in Supplementary Table S1. Samples derived from B16 cells with 2 of 3 loci were carried forward through the pipeline (with others discarded). Finally, samples derived from HAP1 cells were amplified, using primers found in Supplementary Table S1, by touchdown PCR (−0.5 °C per cycle from 72 °C to 60 °C plus 10 additional cycles at 59 °C) at the sgRNA target regions (500 bp downstream and 100-200 bp upstream of the sgRNA sequence). PCR products were separated on a 4% agarose gel and visualized as described in QC-1. All samples derived from HAP1 cells were carried forward through the pipeline.

Next, samples were subjected to step 9 of the genomics pipeline (Supplementary Fig. S5): DNA sequencing. Whole genome sequencing libraries were prepared from 100 to 500 ng of purified and quantified DNA using the Nextera DNA Flex Library Prep Kit (Illumina) following the manufacturer’s instructions. Libraries were loaded at 14 pmol onto an Illumina MiSeq V3 for sequencing at low coverage with 150-bp paired-end sequencing.

Finally, samples were subjected to step 10 of the genomics pipeline (Supplementary Fig. S5): sequencing analysis. Sequencing reads from whole genome DNA sequencing experiments were analyzed as follows, with important pre-alignment, alignment and normalization methods highlighted in Supplementary Fig. S7. First, raw reads were analyzed using FastQC (version 0.11.7) to determine sequencing quality. Next, FASTQ files were trimmed using Trimmomatic (version 0.36) with standard input parameters (Phred scores >33 and read length >20 bp). The resulting trimmed files were aligned to the human reference genome (version hg19) or mouse reference genome (version mm10) using Burrows-Wheeler Aligner (version 0.7.17) “aln” command with standard options. The SAI output files were converted to BAM using Burrows-Wheeler Aligner “sampe” and Samtools (version 1.8) “view” commands. Samtools “sort” was used to sort the resulting BAM files by reference genome position. Next, PCR duplicates were marked and removed using MarkDuplicates (Picard tools). DNA sequencing metrics were determined using Samtools “flagstat”, which provided mapping percentages. Finally, for samples with mapping rate >60%, BAM files were converted to BED format using Bedtools (version 2.25.0) “bamToBed” command.

Sequencing uniformity and copy number variant (CNV) detection were carried out using a protocol similar to that which was developed by Wierman et al.^60^. Briefly, mapped reads from sequenced cells were normalized for sample read depth and were binned into non-overlapping genomic windows containing only uniquely mappable sequences. The read counts for each genomic bin (500 kb per bin) were determined and converted to estimated copy number using a pipeline developed previously^60^. CNV data was visualized by plotting the estimated copy number versus genomic position, and the correlation of estimated copy numbers for DISCO collected samples and bulk DNA. Sequencing uniformity was visualized with a Lorenz curve, by plotting the cumulative fraction of total reads versus cumulative fraction of the genome. Lorenz curves and CNV plots were generated using Python matplotlib. Samples derived from U87/B16 co-cultures were mapped to both the human and mouse genomes to determine the mapping rate efficiency (Supplementary Fig. S9b).

### Transcriptomic analysis of single cells

Aliquots of cell lysate (3 μL) collected from DISCO devices were subjected to step 5 of the transcriptomics pipeline (Supplementary Fig. S10): cDNA synthesis and barcoding. [In spike-in experiments, 60 pg of mouse total RNA (Takara Bio, 636601) was added to each aliquot at this stage.] Briefly, each sample was incubated with 1 μL of capture-oligo (0.25 mM in lysis buffer, Takara, 635013) for 10 min at room temperature. [Each capture-oligo contains a unique molecular identifier (UMI) barcode and a 3ʹ reverse transcriptase start site. The capture-oligos and other custom sequences used here are listed in Supplementary Table S2, largely adapted from the drop-seq protocol^7^]. The lysate/capture-oligo mixture was then mixed with 2.5 μL of Maxima H Minus Reverse Transcriptase solution (ThermoFisher Scientific, EP0752), containing dNTPs (10 mM each, Takara, 639132), RNase Inhibitor (2U/μL Takara, 2313) and a custom template switching primer (1 μM, sequence in Supplementary Table S2) and incubated at 50 °C for 30 min. Samples were then pooled and cDNA was amplified using an Advantage 2 PCR kit (Takara, 639206) as per manufacturer’s instructions, using a custom template-switching hybrid primer (0.25 μM, sequence in Supplementary Table S2).

Next, samples were subjected to step 6 of the transcriptomics pipeline (Supplementary Fig. S10): Nextera XT, bioanalyzer, Qubit. Briefly, cDNA samples were first assessed for quality using a Qubit (for cDNA concentration) and Bioanalyzer (for cDNA size). Samples that had average sizes around 1 kb and Qubit concentrations above 1 ng/μL were carried forward (and others discarded) into RNA-seq libraries using the Nextera XT kit and indices.

Next, samples were subjected to step 7 of the transcriptomics pipeline (Supplementary Fig. S10): RNA sequencing. Samples were run on a MiSeq V3, 2 × 75, with custom settings of read 1 being 36 bp, read 2 being 114 bp, and with a custom sequencing primer spiked in with the Illumina primers (sequence in Supplementary Table S2), with libraries sequenced at 12 pmol.

Finally, samples were subjected to step 8 of the transcriptomics pipeline (Supplementary Fig. S10): sequencing analysis, applying the thresholds from Supplementary Fig. S7. The read-1 fastq files were first parsed to collapse non-unique UMI sequences and then bin fastq files based on the detected cell barcode. All parsing was done using custom scripts and then appended as headers to each corresponding read 2. The read-2 fastq files were then mapped using StarAligner^61^ (v2.6) and filtered for Phred scores >25, read length >100 bp and a concordant mapping rate >60%. The package featureCounts^62^ (Rsubread package, version 3.9) was used to retrieve gene count information. Gene count matrices were then normalized first by gene length (kb), then by library depth to arrive at transcripts per million (TPM) reads. When spike-in data was available, reads were divided by the proportion of reads detected for the spike-in relative to the total reads detected for the sample. Data visualization was implemented in R, with heatmaps formed using gplot^63^ package heatmap2, genes as a function of cumulative TPM plots compilated with ggplot2^64^ and floating pie scatter charts formed and visualized with scatterpie^65^.

### Proteomic analysis of single cells

Aliquots of cell lysate (4 µL) collected from DISCO devices were subjected to step 5 of the proteomics pipeline (Supplementary Fig. S15): reduction, alkylation, and digestion. Briefly, samples were reduced in 5 mM tris(2-carboxyethyl)phosphine (TCEP, final concentration) at 70 °C for 30 min (thermocycler, Bio-Rad), alkylated with 10 mM iodoacetamide (IAA, final concentration) in the dark at room temperature for 30 min. Samples were then digested at 37 °C by mixing with 0.5 μL Lys-C (5 ng/μL) and incubating for 3 h and then mixing with 0.5 μL of trypsin (20 ng/μL) and incubating for 16 h. Finally, digests were quenched by the addition of 0.2 μL of concentrated formic acid.

Next, samples were subjected to steps 6–7 of the proteomics pipeline (Supplementary Fig. S15): chromatography and analysis by mass spectrometry. Briefly, nanoflow reversed-phase liquid chromatography (LC) was performed on an EASY-nLC 1200 ultra-high-pressure system coupled to a Q-Exactive HF-X mass spectrometer equipped with a nano-electrospray ion source (Thermo Fisher Scientific). Peptide digests were loaded onto a 12 cm long fused silica microcapillary column (100-µm i.d., Polymicro Technologies), packed in-house with 1.9 µm diameter ReproSil-Pur C18 120 Å reversed phase particles (Dr. Maisch GmbH). Mobile phases A and B were water with 0.1% formic acid (v/v) and 80/20/0.1% ACN/water/formic acid (v/v/v), respectively. Peptides were separated at a constant flow rate of 250 nL/min with a linear gradient of 3–30% mobile phase B for 90 min, followed by a linear increase from 30–45% mobile phase B for 20 min, then a linear increase from 45–95% mobile phase B for 1 min and a 14-min plateau at 95% mobile phase B before re-equilibration. Standard shotgun LC-MS experiments were performed with a data-dependent top10 method, using a full MS scan range of *m*/*z* 375–1575 at a resolution of 60,000 at *m*/*z* 200 with an automatic gain control (AGC) target of 5 × 10^5^ ions and maximum injection time of 50 ms. Precursor ions with charges of +2 to +6 were isolated with an *m*/*z* window of 2 and fragmented by high energy dissociation with a collision energy of 27% at a resolution of 15,000 at *m*/*z* 200. MS/MS scans were performed in the Orbitrap with the AGC target, and injection time set to 5 × 10^4^ and 250 ms respectively. Previously targeted precursors were excluded from re-sequencing for 20 s.

Finally, samples were subjected to step 8 of the proteomics pipeline (Supplementary Fig. S15): analysis, applying the thresholds from Supplementary Fig. S7. Raw MS files were analyzed by MaxQuant (version 1.6.4.0)^66^. MS/MS spectra were searched against human protein database from Uniprot, allowing for variable modifications of methionine oxidation and N-terminal acetylation and fixed cysteine carbamidomethylation. The specific proteolytic enzyme was trypsin. The minimum peptide length was six amino acids and maximum peptide mass was 4600 Da. The allowed missed cleavages for each peptide was 2. Both peptides and proteins were filtered with a maximum false discovery rate (FDR) of 0.01. The default settings of Maxquant were used for all unmentioned parameters. PANTHER^67^, REVIGO^68^, and Cytoscape^69^ were used for biological function analysis.

### Genomic analysis of single CRISPR-modified cells

HAP1 cells were mutagenized using CRISPR, with sgRNA sequence (GCACTTAAATATAGATCCGG), as described previously^49^. The CRISPR modified HAP1 cells were treated with a modified version of the genomics analysis pipeline (Supplementary Fig. S5). After steps 1–2 (described above), cells were subjected to an automated immunofluorescent staining protocol. Each sub-step comprised loading a reagent into a reservoir, dispensing eight 2-μL droplets onto the array of actuation electrodes, driving each droplet to the virtual microwell containing fixed cells, incubating the device with virtual microwells at a given temperature and duration, and then driving the droplets to a waste reservoir. The reagents and incubation conditions are indicated in the following list. (sub-step i) reagent: DPBS with 0.05% (v/v) F68; incubation: none (wash). (sub-step ii) reagent: 5% (v/v) FBS with 0.05% (v/v) Tween-20 in DPBS; incubation: room temperature, 1 h (block). (sub-step iii) reagent: anti-human CD47^−^APC (clone CC2C6, BioLegend, 323104) diluted 1:250 in primary dilution solution [1% (v/v) FBS with 0.05% (v/v) Tween-20 in DPBS]; temperature: 4 °C, overnight (primary label). (sub-step iii) reagent: Hoechst 33342 diluted 1:5,000 in DPBS with 0.05% (v/v) F68; temperature: room temperature, 10 min (nuclear label) (sub-steps v and vi): repeats of sub-step i (wash). At the completion of the labeling protocol, samples were imaged to identify WT (Hoechst^+^ CD47^+^) and KO (Hoechst^+^ CD47^−^) cells, with CD47 labeling intensity analysis performed using Zen blue edition (Zeiss). Prism 7 by GraphPad was used to perform statistical analysis, and a one-way ANOVA with a Tukey’s multiple comparison test with *α* = 0.05 was used to determine significance. Samples were then subjected to genomics analysis pipeline (Supplementary Fig. S5) steps 3 and 4 to select and lyse individual cells of each phenotype, and steps 5–8 (WGA, QC-1, purification, QC-2). Finally, in place of steps 9–10, samples were evaluated by Sanger sequencing. Briefly, a 7 µL aliquot of purified and quantified DNA (50 ng total) was mixed with 1 µL of the forward (GCCCTGATGACGTCCTGATT) or reverse (TATGTAGAGGCCAGGGATGC) primer, amplified, and sequenced using an ABI 3730 XL. Variability in the genotype for KO cells was determined by aligning the sequencing data to that of the WT cells.

### Statistics and reproducibility

Images in Figs. 1c, 2d, 4d, 4e, 6a, b, S6a, and S9a were selected as representatives of replicate results. Similar results were obtained in at least 3 independent experiments in all cases.

### Reporting summary

Further information on research design is available in the Nature Research Reporting Summary linked to this article.

## Supplementary information

Supplementary Information

Supplementary Movie 1

Supplementary Movie 2

Supplementary Data 1

Supplementary Data 2

Description of Additional Supplementary Files

Reporting Summary

## Data Availability

All DNA sequencing and RNA sequencing data described in this paper are available under BioProject accession PRJNA640377 and PRJNA640061, respectively. All mass spectrometry proteomics data described in this paper have been deposited to the ProteomeXchange Consortium (http://proteomecentral.proteomexchange.org) via the PRIDE partner repository^70^ with identifier PXD019958. Source data are provided with this paper.

## Source data

Source Data

## Code availability

All scripts used to process images for AI, scripts to process genomic and transcriptomic data, and software used to run DISCO are found at http://microfluidics.utoronto.ca/gitlab/DISCO.
