# Supplementary material for: Digital microfluidic isolation of single cells for -Omics
Source: Nat Commun. 2020 Nov 11;11:5632. doi: 10.1038/s41467-020-19394-5 (PMC7658233; doi:10.1038/s41467-020-19394-5)
Supplement: Supplementary file 1 — Supplementary Information [file 41467_2020_19394_MOESM1_ESM.docx]

**Supplementary Information** for

Digital Microfluidic Isolation of Single Cells for -Omics

Julian Lamanna,^1,2^* Erica Y. Scott,^1,2^* Harrison S. Edwards,^2,3^* M. Dean Chamberlain,^1,2^ Michael D. M. Dryden,^1^ Jiaxi Peng,^1^ Barbara Mair,^2^ Adam Lee,^3^ Calvin Chan,^1^ Alexandros A. Sklavounos,^1,2^ Austin Heffernan,^2^ Farhana Abbas,^1,2^ Charis Lam,^1^ Maxwell E. Olson,^1^ Jason Moffat,^2,3,4^ and Aaron R. Wheeler^1,2,3^†

^1^ Department of Chemistry, University of Toronto, 80 St. George Street, Toronto, Ontario M5S 3H6, Canada

^2^ Donnelly Centre for Cellular and Biomolecular Research, University of Toronto, 160 College Street, Toronto, Ontario M5S 3E1, Canada

^3^ Institute of Biomaterials and Biomedical Engineering, University of Toronto, 164 College Street, Toronto, Ontario, M5S 3G9, Canada

^4^ Department of Molecular Genetics, University of Toronto, Toronto, Ontario, Canada

†Corresponding author. Email: [aaron.wheeler@utoronto.ca](mailto:aaron.wheeler@utoronto.ca)

*These authors contributed equally


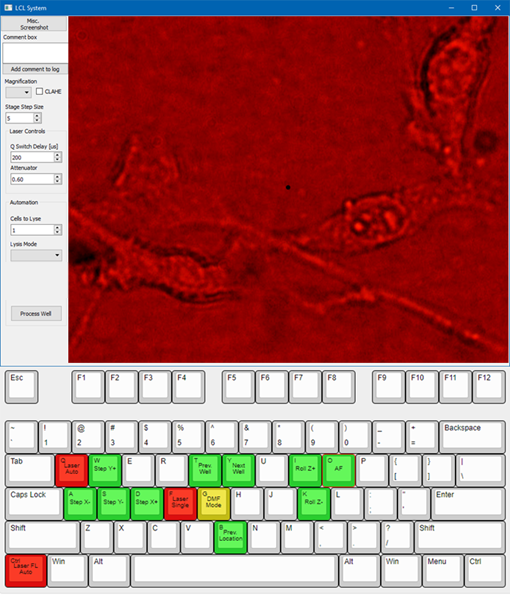


Supplementary Figure S1. **Custom graphical user interface (GUI, top) and hot keys (bottom) for the DISCO system.** The GUI features a live video-feed of the portion of the device being interrogated, space for the user to select key parameters related to magnification, and laser controls for manual- or AI-driven lysis. The schematic highlights hot keys that are dedicated to laser lysis (red), DMF mode (yellow), and DISCO device movement (green).


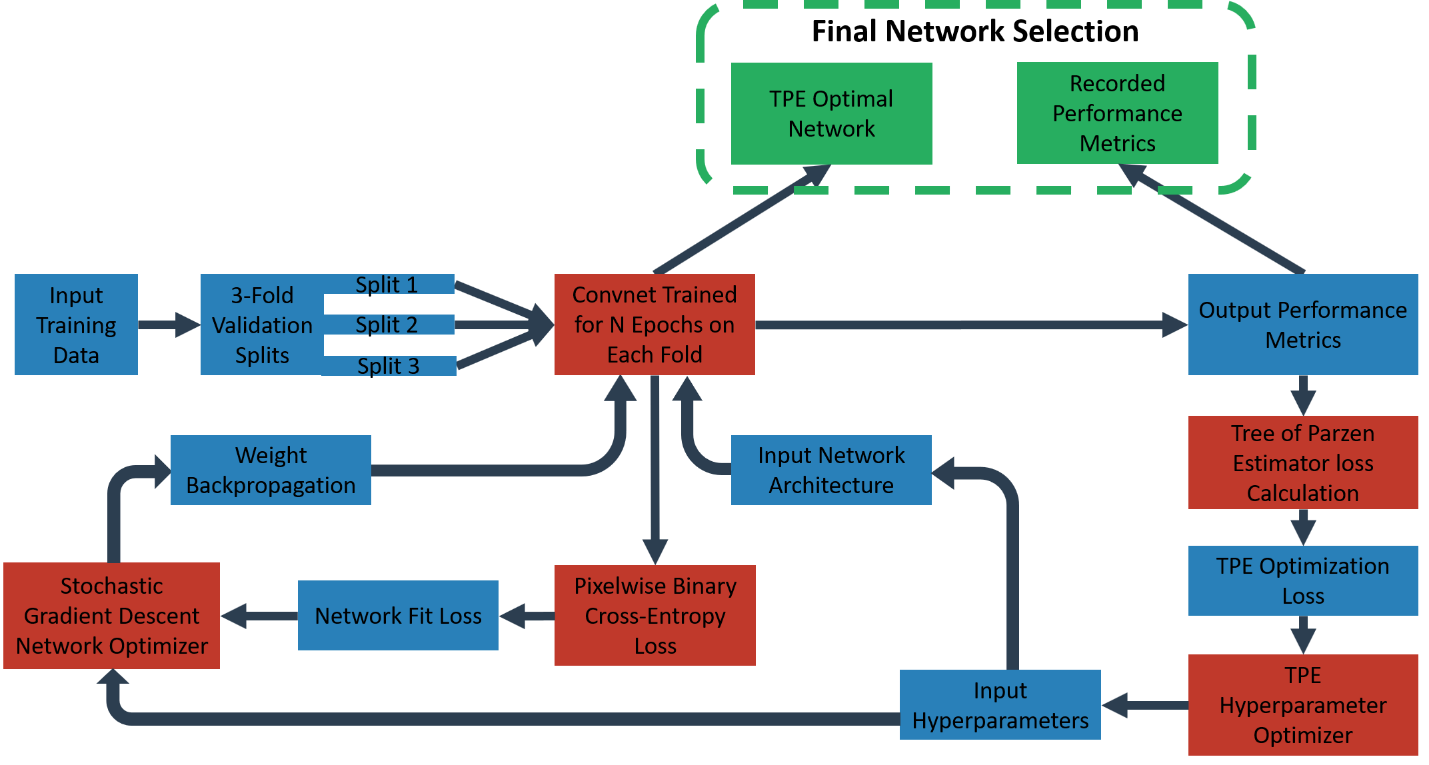


Supplementary Figure S2. **Hyperparameter optimization scheme for candidate models for AI control of DISCO.** Flow diagram illustrating the process for model hyper-parameter optimization. Model hyperparameters corresponding to the labels in Fig. 2a in the main text are (initially) randomly chosen. The input training data is split into three different training/validation splits at a ratio of 2/3 training data to 1/3 validation data. The model with the chosen hyperparameters is trained for five epochs on each of the three training/validation splits, with re-initialization at each split. The model performance metrics are generated via evaluation of the validation data and the metrics from the three different splits are averaged. The metrics are then passed to a Tree of Parzen Estimator (TPE) that generates a probability distribution for well-performing model hyperparameters. The loss function chosen for optimization via the TPE algorithm is the negative sum of the area under curve (AUC) and the average precision (AP).


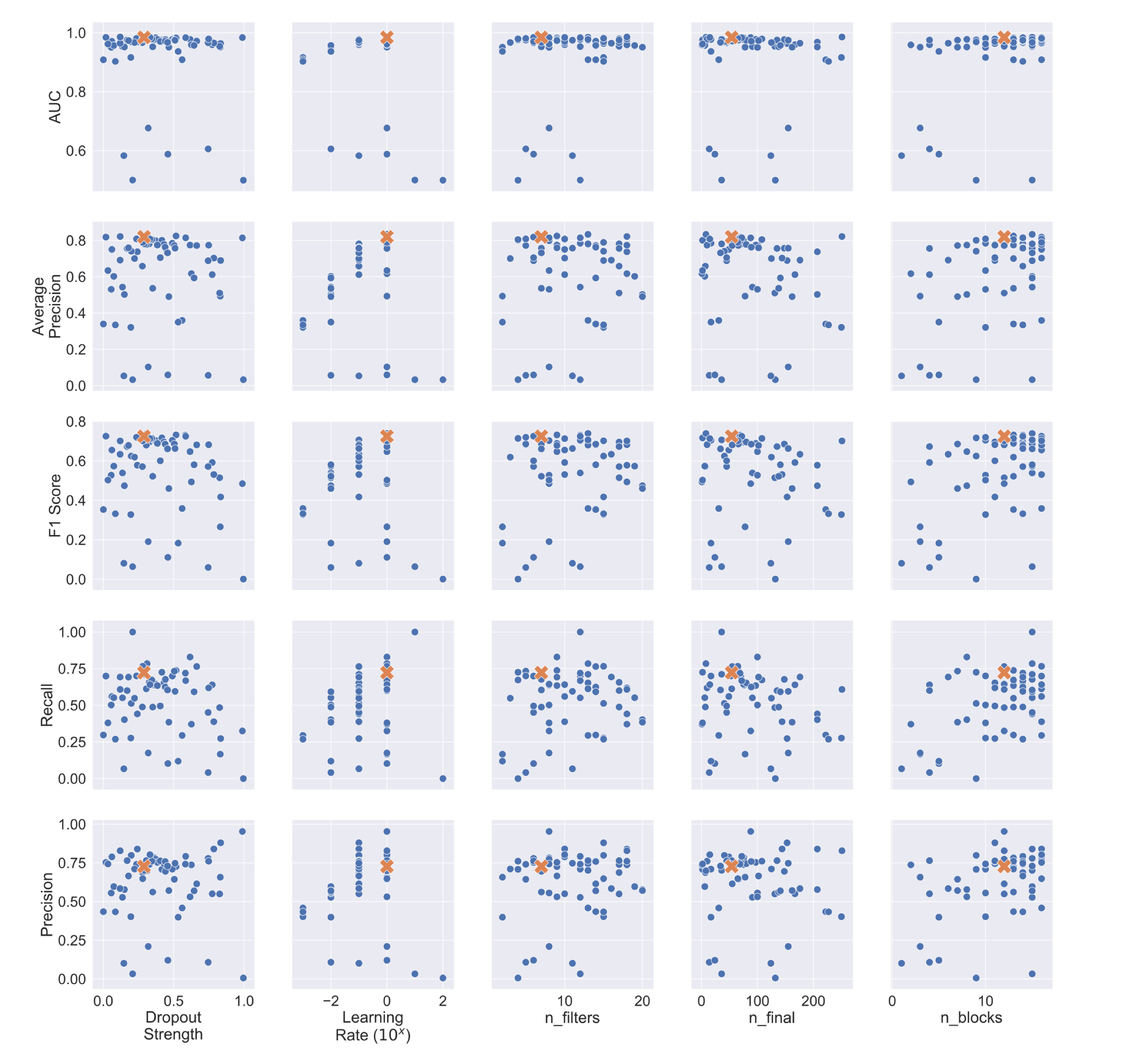


Supplementary Figure S3. **Performance of candidate models for AI control of DISCO.** Plots of performance metrics (y-axes) as a function of hyperparameters (x-axes) for approximately 100 models trained (blue dots) on the CNN. The orange x indicates the ultimate model selected for AI control of DISCO. The performance metrics include area under curve (row 1), average precision (row 2), F1 score (row 3), Recall (row 4), and Precision (row 5). The hyperparameters include dropout strength (column 1), learning rate (column 2), number of filters in intermediate blocks (column 3), the number filters in the final layer (column 4) and the number of intermediate blocks (column 5).


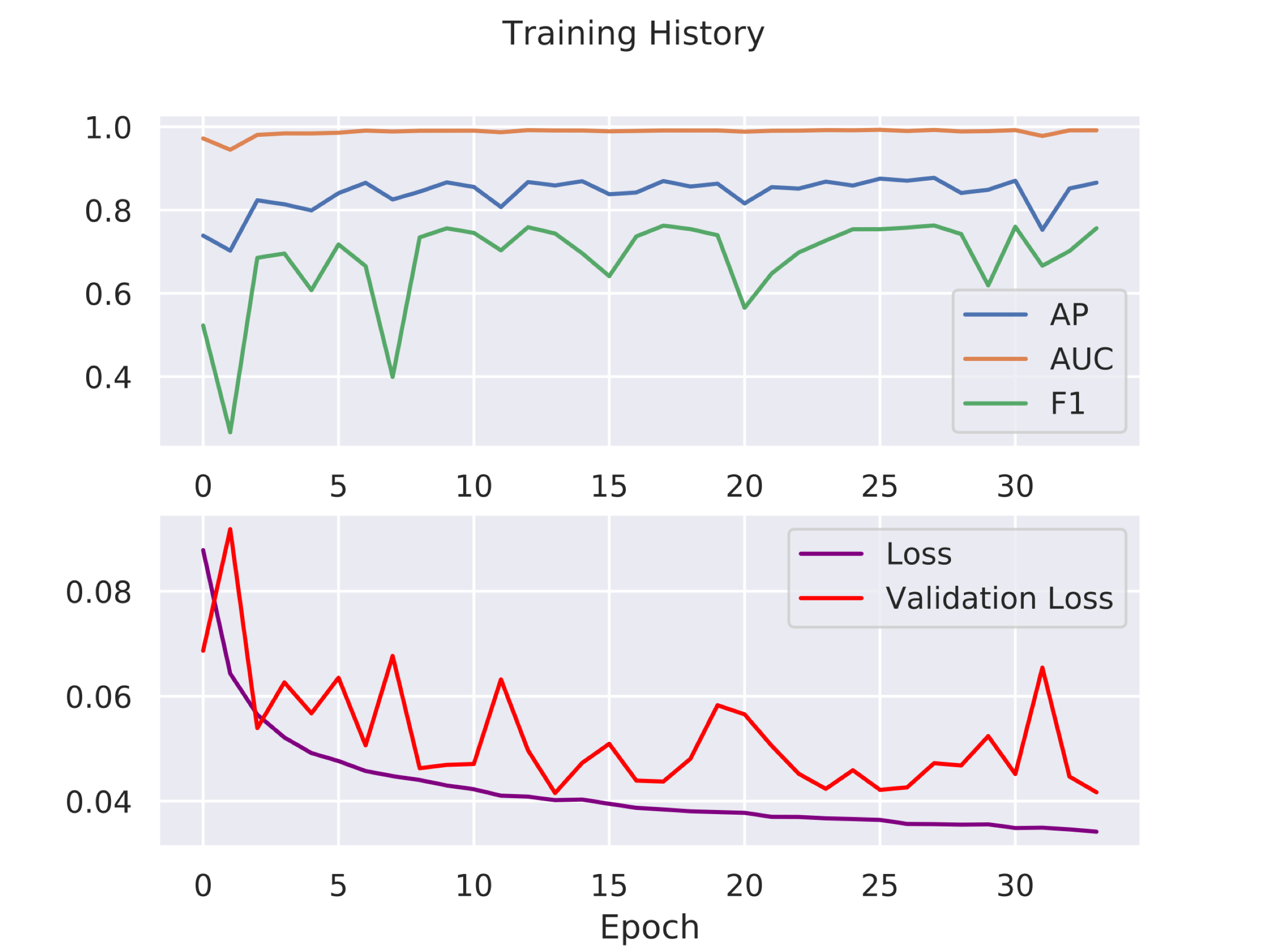


Supplementary Figure S4. **DISCO AI model training history**. Plots showing performance metrics (top) including average precision (blue), area under curve (orange), and F1 score (green) and loss (bottom) including loss (purple) and validation loss (red) as a function of epoch during training of the hyper-optimized model that was ultimately selected for DISCO AI control.


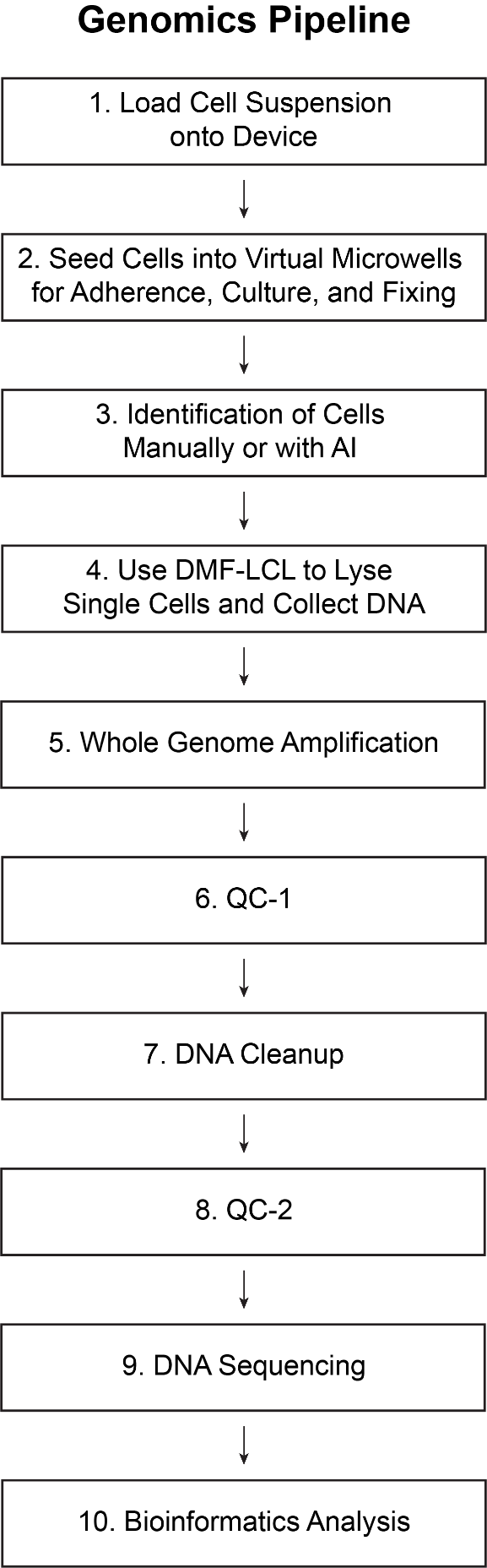


Supplementary Figure S5. **Custom pipeline for single-cell genome sequencing.** Steps 1-4 are implemented by DISCO; steps 5-10 are described in detail in the methods section of the main text.


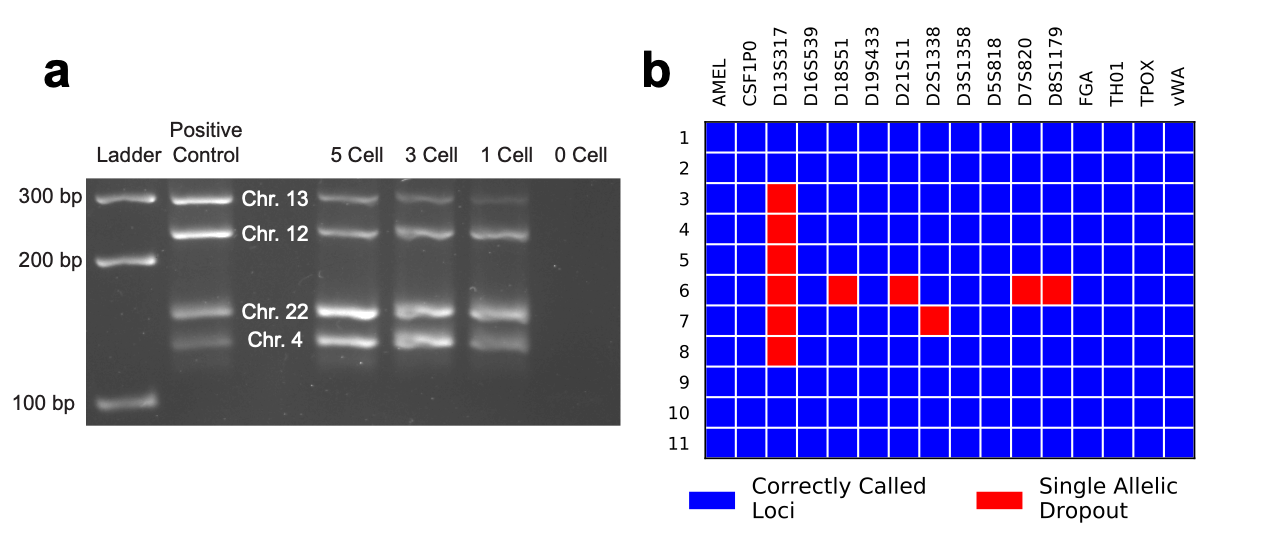


Supplementary Figure S6. **Results from genome analysis quality control tests 1 and 2 (QC-1 and QC-2).** U87 cell monocultures were loaded onto DISCO devices, where they were fixed, targeted (0, 1, 3, or 5 cells per droplet), lysed, and amplified according to the genome sequencing pipeline (Supplementary Fig. S5). (a) Image of an agarose gel after electrophoretic separation of PCR products from targeted amplification at 4 loci on chromosomes 4, 12, 13, and 22 in QC-1. The sample in the left lane (positive control) was a human genome standard. (b) Table illustrating the results of short tandem repeat polymerase chain reaction (STR-PCR) analysis of 11 single cells (rows) measured across 16 genomic loci (columns) in QC-2. Blue and red squares correspond to correct/incorrect assignment of genomic loci, respectively.


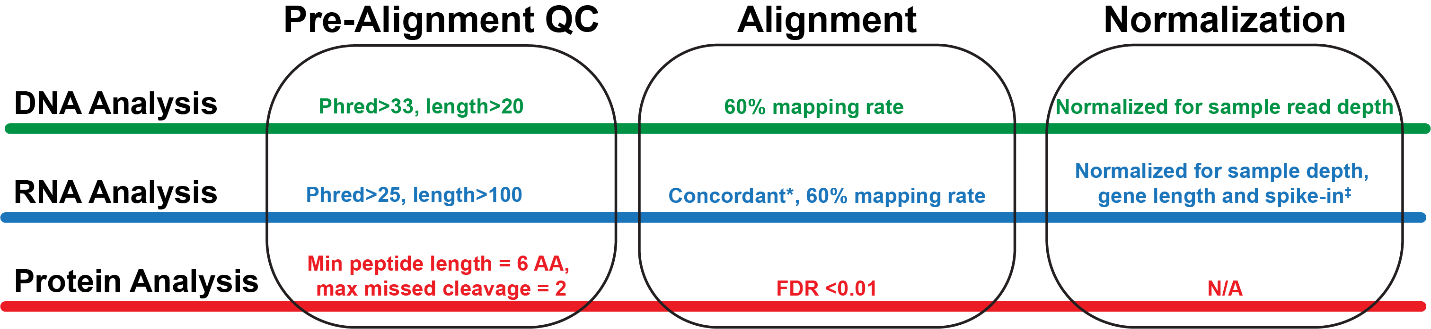


Supplementary Figure S7. **Bioinformatics thresholds for multi-omics analyses.** Pre-alignment quality control (QC; including Phred quality score, minimum sequence/peptide length), alignment criteria [including genome mapping rate and false discovery rate (FDR)] and normalization measures are shown for single cell DNA and RNA analyses. *Concordant reads refer to paired-end reads mapping together and ^‡^spike-in refers to control RNA spiked in as a measure of mRNA capture efficiency.


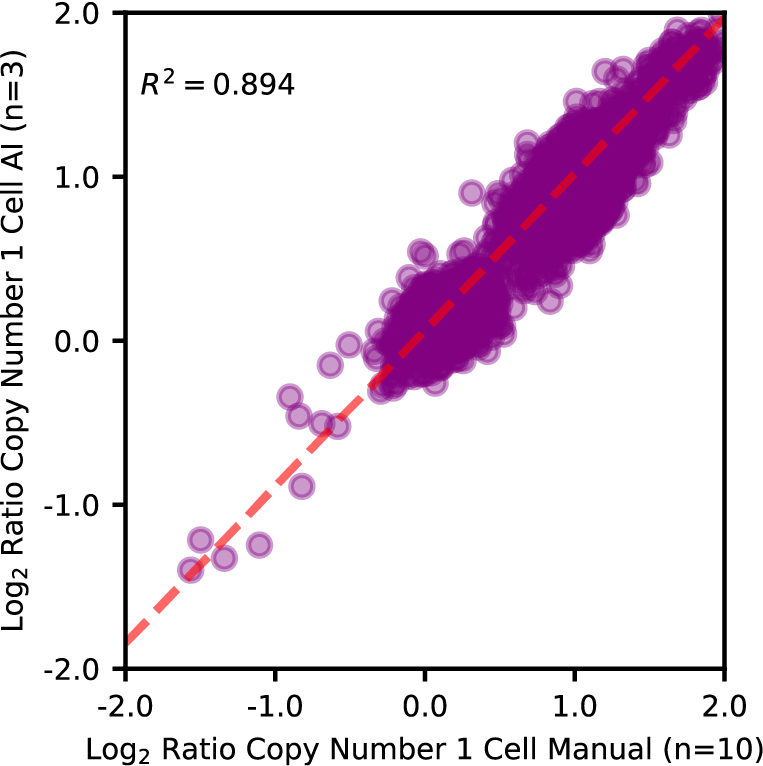


Supplementary Figure S8. **Genome** **correlation data for manual versus AI driven single cell lysis.** U87 cell monocultures were loaded onto DISCO devices, where they were fixed, targeted (1 cell per droplet), lysed, and amplified according to the genome sequencing pipeline (Supplementary Fig. S5). Log_2_ correlation plot of copy numbers for cells targeted using AI (n=3) and manually (n=10). Each purple circle represents the estimated copy number within a specific bin of size 500kb. A linear least squares regression fit (dashed red line) and coefficient of determination (R^2^=0.894) are shown.


Supplementary Figure S9. **Evaluation of DISCO target-specificity in heterogeneous samples.** Co-cultures of eGFP-expressing U87 cells and tdTomato-expressing B16 cells were loaded onto DISCO devices, where they were fixed, targeted (0, 1 or 5 cells per droplet), lysed, and amplified according to the genome sequencing pipeline (Supplementary Fig. S5). For comparison, bulk samples co-cultures (~1,000 cells) were also evaluated (foregoing the pipeline, with no amplification). (a) Image of an agarose gel showing the separation of PCR products after amplification using eGFP (G) and tdTomato (T) primers for DNA collected from 0 U87 cells (sample 0, left) or 5 U87 cells (samples 1-3, right). (b) Plot of mapping-rate efficiency to the human genome (left) and mouse genome (right) for DNA extracted from human-derived U87 cells (green) and mouse-derived B16 cells (red). Single-cell analyses are in light-green/red and bulk analyses are in dark green/red. Data shown are averages of replicate analyses (individual replicates represented by black, open circles): n=3 and n=4 single U87 and B16 cells, and n = 3 and n = 2 of bulk aliquots of U87 and B16 cells, respectively. Error bars represent ±1 SEM.


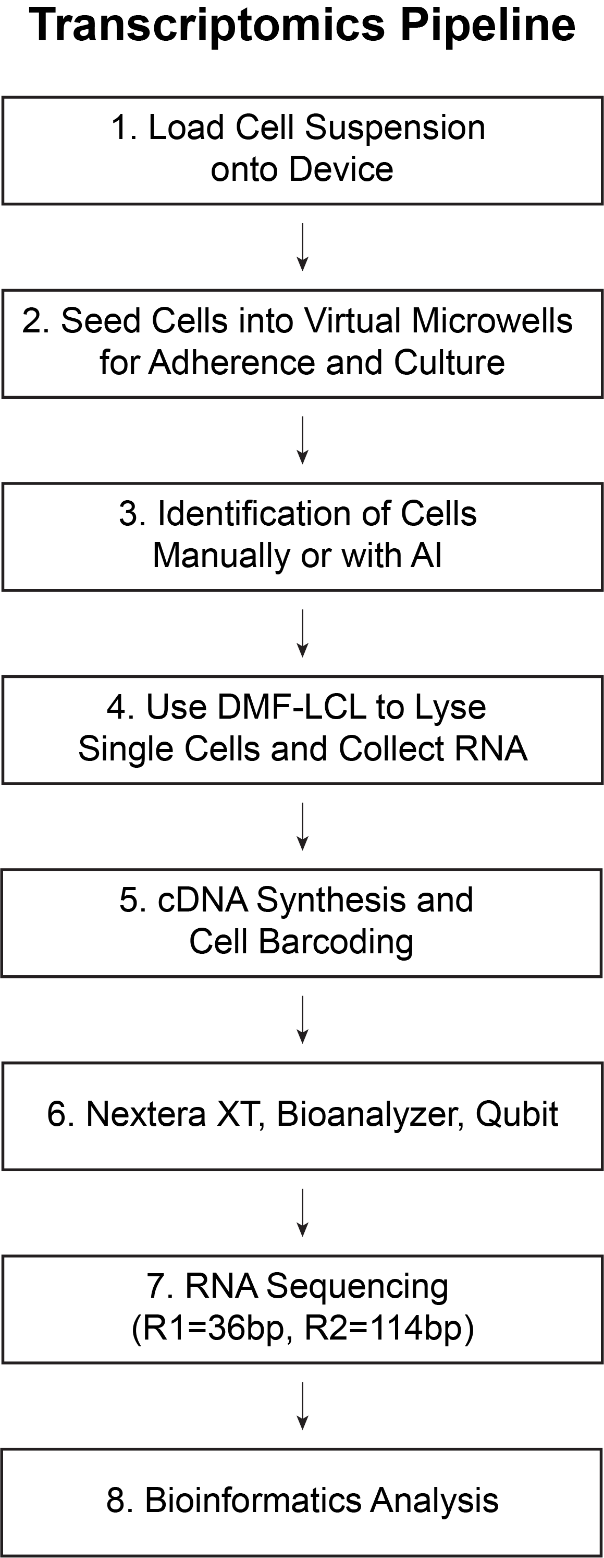


Supplementary Figure S10. **Custom pipeline for single-cell transcriptome sequencing.** Steps 1‑4 are implemented by DISCO and are identical to the analogous steps for genome sequencing, except with no cell fixation. Steps 5-8 are described in detail in the methods section of the main text. Step 6 utilizes the Bioanalyzer and Qubit to produce a Nextera XT prepared RNA-seq library that is approximately 1 nM for sequencing. In Step 7, R1 and R2, refer to Read 1 and Read 2 on the Illumina Miseq, with the number of base pairs (bp) denoted for each.

Supplementary Figure S11. **Effect of cell input number on DISCO transcriptome analysis.** B16 cell monocultures were loaded onto DISCO devices, where they were targeted (0, 1, or 2 cells per droplet), lysed, and analyzed according to the transcriptome sequencing pipeline (Supplementary Fig. S10). For comparison, bulk samples of monocultures (~1,000 cells) were also evaluated (foregoing the pipeline). (a) Plot of the numbers of genes detected as a function of number of transcripts detected (quantified as transcripts per million reads, TPM) for 0- (red), 1- (green), and 2-cell (blue) samples, as well as bulk samples (brown). Results from two replicates are down for each condition, with transcriptome coverage represented by the diameter of the marker. (b) Heat map of the average gene expression level (blue-low, yellow-high, purple-zero) of the 82 genes with most consistent and robust expression (sum TPM >80, SEM<1) across samples of 1-cell, 2-cell and bulk. The white trace labeled "density" in the legend represents the distribution of the different expression levels in the data-set.

Supplementary Figure S12. **Effect of manual vs. AI selection of cells for DISCO transcriptome analysis**. B16 or U87 cell monocultures were loaded onto DISCO devices, where they were targeted (1 or 2 cells per droplet), lysed, and analyzed according to the transcriptome sequencing pipeline (Supplementary Fig. S10). For comparison, bulk samples of monocultures (~1,000 cells) were also evaluated (foregoing the pipeline). Plot of the numbers of genes detected as function of number of transcripts detected (quantified as TPM) for U87 (green) and B16 (red) cells (light-1 cell, med-2 cells, dark-bulk). Two results each are shown for DISCO-AI (circles), bulk (squares), and DISCO-manual (triangles) samples, with transcriptome coverage represented by the size of the marker.


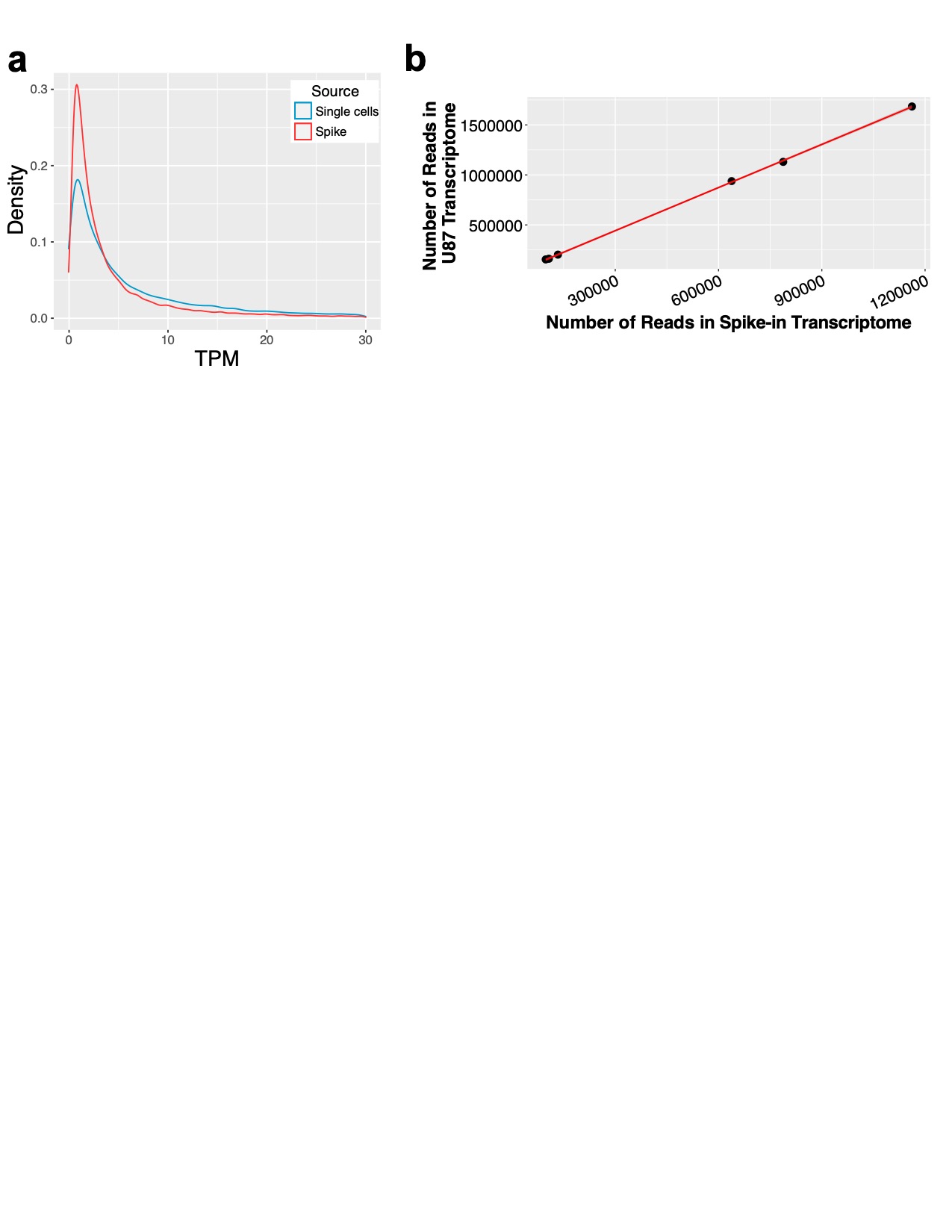


Supplementary Figure S13. **Technical noise and biological variation in DISCO single cell transcriptome data.** U87 cell monocultures were loaded onto DISCO devices, where they were targeted (1 or 2 cells per droplet), lysed, and analyzed according to the transcriptome sequencing pipeline (Supplementary Fig. S10), with mouse transcriptome standard spiked in to each sample in step 5. For comparison, bulk samples of monocultures (~1,000 cells) were also evaluated (also with spiked standard). (a) Plot of the density of reads as a function of the normalized, averaged number of transcripts (as transcripts per million, TPM) from three single U87 cells (blue) representing biological variation + technical noise and the corresponding spike-in data for mouse transcriptome standard (red) representing technical noise only. (b) Plot of the number of U87 (human) transcriptome reads as a function of the number of spike-in mouse transcriptome reads for 1-cell, 2-cell, and bulk samples (black markers) with linear fit (red line) with slope = 1.44. The strong correlation (R^2^ = 0.999, p<0.0001, linear regression) suggests that technical noise is predictable, and that it may be possible to normalize to emphasize biological variation in the future.


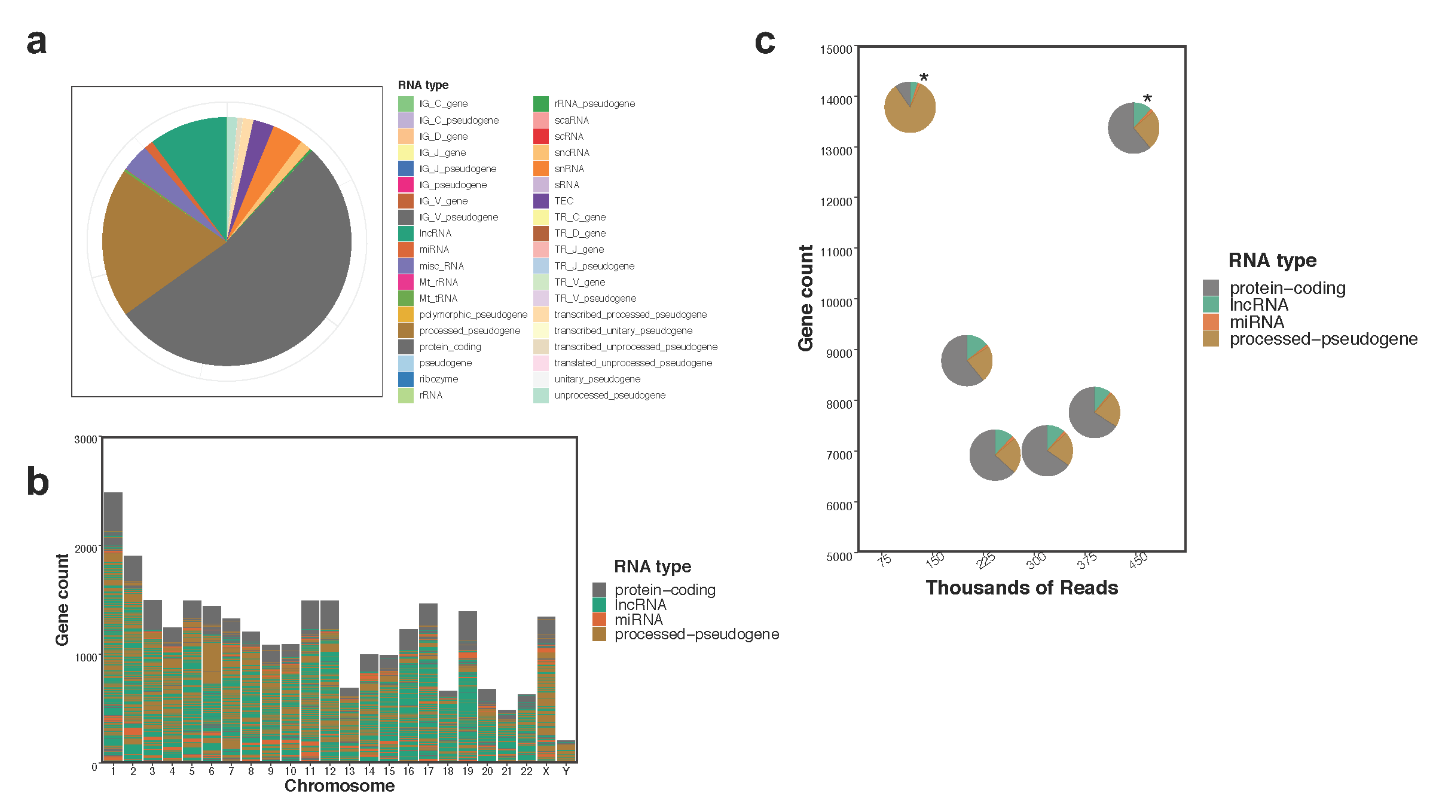


Supplementary Figure S14. **DISCO-based** **analysis of gene type, content, and coverage for U87 cells.** U87 cell monocultures were loaded onto DISCO devices, where they were targeted (1 cell per droplet), lysed, and analyzed according to the transcriptome sequencing pipeline (Supplementary Fig. S10). For comparison, bulk samples of monocultures (~1,000 cells) were also evaluated (foregoing the pipeline). (a) Pie chart showing consolidated gene expression from n=4 representative single U87 cells binned into the type of RNA the expression originated from. (b) Plot of protein-coding (gray), lncRNA (green), miRNA (orange) and processed pseudogenes (yellow) as a function of chromosome for the data-set in (a). (c) Plot of the number of genes detected per cell as a function of aligned reads for the data-set from (a) broken-out by cell compared to n=2 bulk samples (labelled with asterisks). Each marker is a pie-chart broken into the categories from (b).


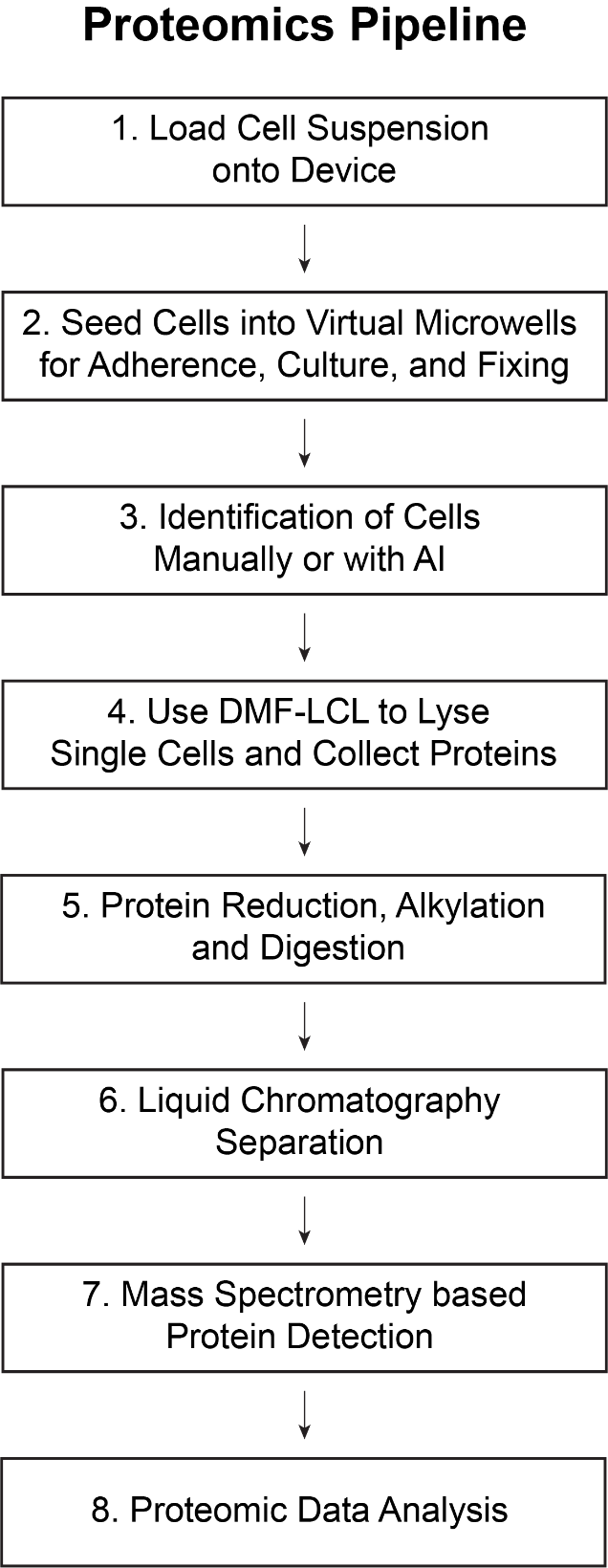


Supplementary Figure S15. **Custom pipeline for single-cell proteome sequencing.** Steps 1-4 are implemented by DISCO; steps 5-8 are described in detail in the methods section of the main text.


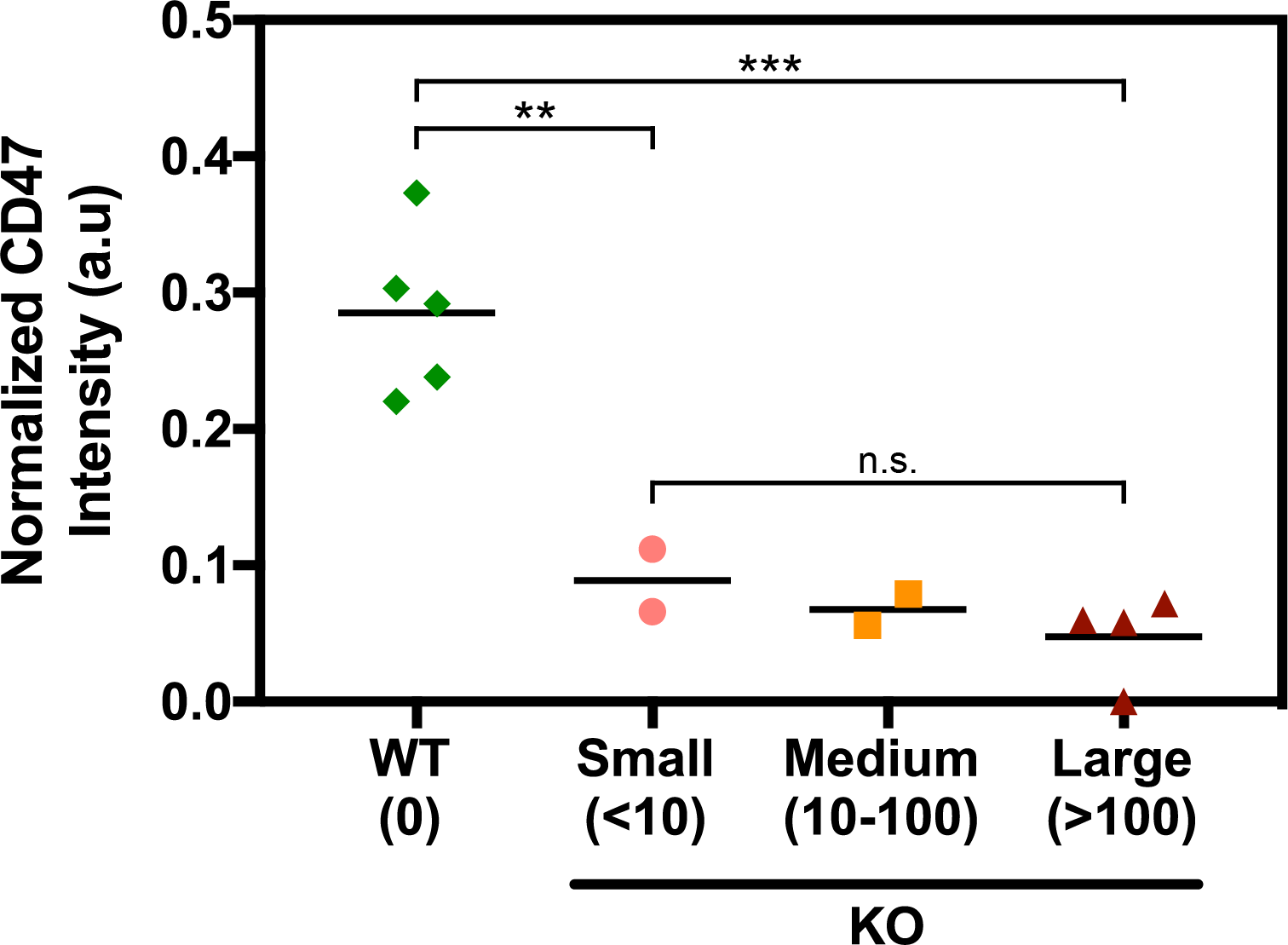


Supplementary Figure S16. **Phenotype to genotype analysis of CRISPR modified cells using DISCO.** Normalized CD47 staining intensity in immunofluorescent images generated using DISCO. Cells were divided into 4 bins on the basis of Sanger sequencing results, where each marker refers to an individual cell: WT (0 bp, green diamonds, n=5), KO-small (<10 bp, pink circles, n=2), KO-medium (10-100 bp, orange squares, n=2), or KO-large (>100 bp, maroon triangles, n=4). Horizontal black bars represent the mean normalized labeling intensities. ** p = 0.0029, *** p = 0.0001, n.s. p = 0.7300, determined by one-way ANOVA with a Tukey’s multiple comparison test with α = 0.05.

Supplementary Table S1. **Primers used for single-cell genome sequencing pipeline (Supplementary** **Fig. S5)**.

| Name | Forward Sequence | Reverse Sequence |
| --- | --- | --- |
| Chromosome 4 (Human) | GCAAAATCCATACCCTTTCTGC | TCTTTCCCTCTACAACCCTCTAACC |
| Chromosome 12 (Human) | TTTGATGTTAGGACACGCTGAAA | AAAAACGGAAGAAGTCTCTTGGC |
| Chromosome 13 (Human) | GTCAGAAGACTGAAAACGAAGCC | GCTTGCCACACTCTTCTTCAAGT |
| Chromosome 22 (Human) | GCTGTTAGAGCTTTTATTGCAGC | CTAGAAATTTCTGCATAAACCAACC |
| GFP | GAAGCAGCAGCACTTCTTCAA | AAGTCGATGCCCTTCAGCTC |
| tdTomato | AGCAAGGGCGAGGAGGTCATC | CCTTGGAGCCGTACATGAACTGG |
| Chromosome 3 (Mouse) | CTAGGCCACAGAATTGAAAGATCT | GTAGGTGGAAATTCTAGCATCATCC |
| Chromosome 6 (Mouse) | CTCCCAACCCCAGAGGTAGT | CTATAGGGCCTGGGTCAGTG |
| Chromosome 15 (Mouse) | AGAGTGACCAGGCTCAGGAT | AACCGTCAATGCCCCTGCGT |
| CD47 | GCCCTGATGACGTCCTGATT | TATGTAGAGGCCAGGGATGC |

Supplementary Table S2. **Custom-capture oligos and primers used for single-cell transcriptome sequencing pipeline (Supplementary** **Fig. S10).**

| Name | Sequence |
| --- | --- |
| Cell 1 Barcode | /5Biosg/TTTTTAAGC+AGTGGT+ATCAAC+GCAGAGTA+CNNNNN+ TAGCTCGTGCCT +GNNNNNNNNTTTTTTTTTTTTTTTTTTTTTTTTTTTTTTN |
| Cell 2 Barcode | /5Biosg/TTTTTAAGC+AGTGGT+ATCAAC+GCAGAGTA+CNNNNNNN+CTATGTGTCAC+ANNNNNNNNTTTTTTTTTTTTTTTTTTTTTTTTTTTTTTN |
| Cell 3 Barcode | /5Biosg/TTTTTAAGC+AGTGGT+ATCAAC+GCAGAGTA+CNNNNNNNN+GCGCGTAG+CTTGNNNNNNNNTTTTTTTTTTTTTTTTTTTTTTTTTTTTTTN |
| Cell 4 Barcode | /5Biosg/TTTTTAAGC+AGTGGT+ATCAAC+GCAGAGTA+CNNNNNNNNN+GCACACCTGC+TCNNNNNNNNTTTTTTTTTTTTTTTTTTTTTTTTTTTTTTN |
| Cell 5 Barcode | /5Biosg/TTTTTAAGC+AGTGGT+ATCAAC+GCAGAGTA+CNNNNNNNNNN+AGACATGAGA+GTNNNNNNNNTTTTTTTTTTTTTTTTTTTTTTTTTTTTTTN |
| Template Switching oligo | AAGCAGTGGTATCAACGCAGAGTACATrGrG+G |
| Custom Read 1 (sequencing) | GCCTGTCCGCGGAAGCAGTGGTATCAACGCAGAGTAC |
| LD PCR oligo | AAGCAGTGGTATCAACGCAGAGT |
| P5 Illumina Oligo | AATGATACGGCGACCACCGAGATCTACACGCCT\|GTCCGCGGAAGCAGTGGTATCAACGCAGAGT* A*C |

Supplementary Table S3. **Phenotype and genotype of single CRISPR-modified HAP1 cells analyzed by DISCO.** Phenotypes were determined by immunofluorescence microscopy, and genotypes were determined by Sanger sequencing, represented as nucleotides missing relative to WT sequence (Δ Nucleotide).

| Sample | Phenotype | Δ Nucleotide | CD47 Normalized Intensity (a.u) |
| --- | --- | --- | --- |
| 1 | WT | 0 | 0.238 |
| 2 | KO | Major Deletion | 0.072 |
| 3 | WT | 0 | 0.292 |
| 4 | KO | -115 | 0.000 |
| 5 | WT | 0 | 0.303 |
| 6 | WT | 0 | 0.373 |
| 7 | KO | Major Deletion | 0.058 |
| 8 | KO | -1 | 0.112 |
| 9 | KO | -28 | 0.056 |
| 10 | WT | 0 | 0.220 |
| 11 | KO | -77 | 0.079 |
| 12 | KO | -5 | 0.066 |
| 13 | KO | Major Deletion | 0.060 |
