# Supplementary material for: Digital microfluidic isolation of single cells for -Omics
Source: Nat Commun. 2020 Nov 11;11:5632. doi: 10.1038/s41467-020-19394-5 (PMC7658233; doi:10.1038/s41467-020-19394-5)
Supplement: Supplementary file 6 — Description of Additional Supplementary Files [file 41467_2020_19394_MOESM6_ESM.pdf]

Title: Supplementary Data 1.
Description: Gene annotations and corresponding expression values for single cell transcriptome analysis.

Title: Supplementary Data 2.
Description: List of proteins identified by single cell proteomics analysis.

Title: Supplementary Movie 1.
Description: Adjacent cell separation using DISCO. The movie illustrates sub-step (ii) of the cell selection and lysis procedure (see methods section in the main text) at 100X magnification. The clip depicts the isolation, using multiple low powered laser shots, of a single fixed U87 cell that is adjacent (touching) to a neighbouring fixed U87 cell into a droplet.

Title: Supplementary Movie 2.
Description: Lysis and collection of cell contents using DISCO. The movie illustrates sub-steps (ii) and (iii) of the cell selection and lysis procedure (see methods section in the main text) at 100X magnification. There are two clips that run simultaneously, synchronized in time. The clip on the left depicts the view through the microscope objective as a single fixed U87 cell is lysed into a droplet. The clip on the right depicts a view collected from above, as the droplet containing the cell contents is collected for analysis.
