# Supplementary figures and images for: Digital microfluidic isolation of single cells for -Omics
Source: Nat Commun. 2020 Nov 11;11:5632. doi: 10.1038/s41467-020-19394-5 (PMC7658233; doi:10.1038/s41467-020-19394-5)

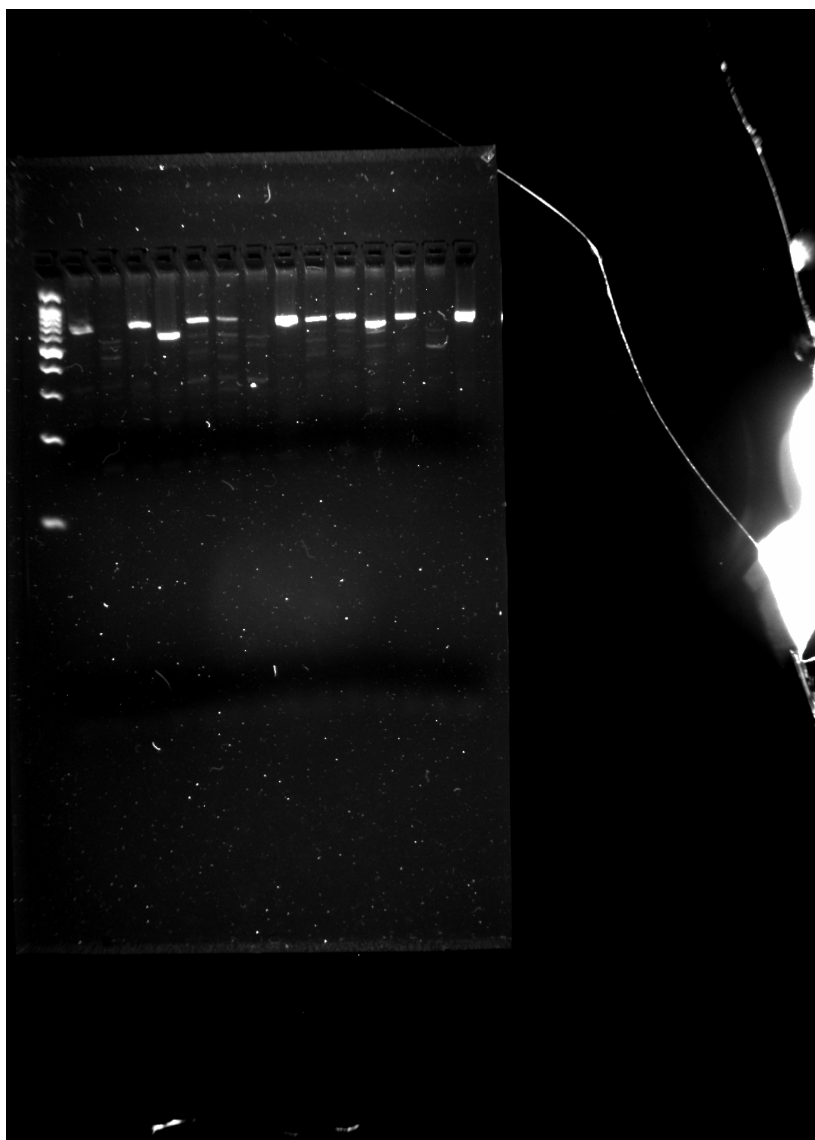

Supplement: Supplementary file 8 — Source Data [file 41467_2020_19394_MOESM8_ESM.zip › Source Data/Figure 6c, S6a, S9a_Gels/CRISPR gel.pdf]

good 5,3,1 cell 2018-10-17 14hr 08min

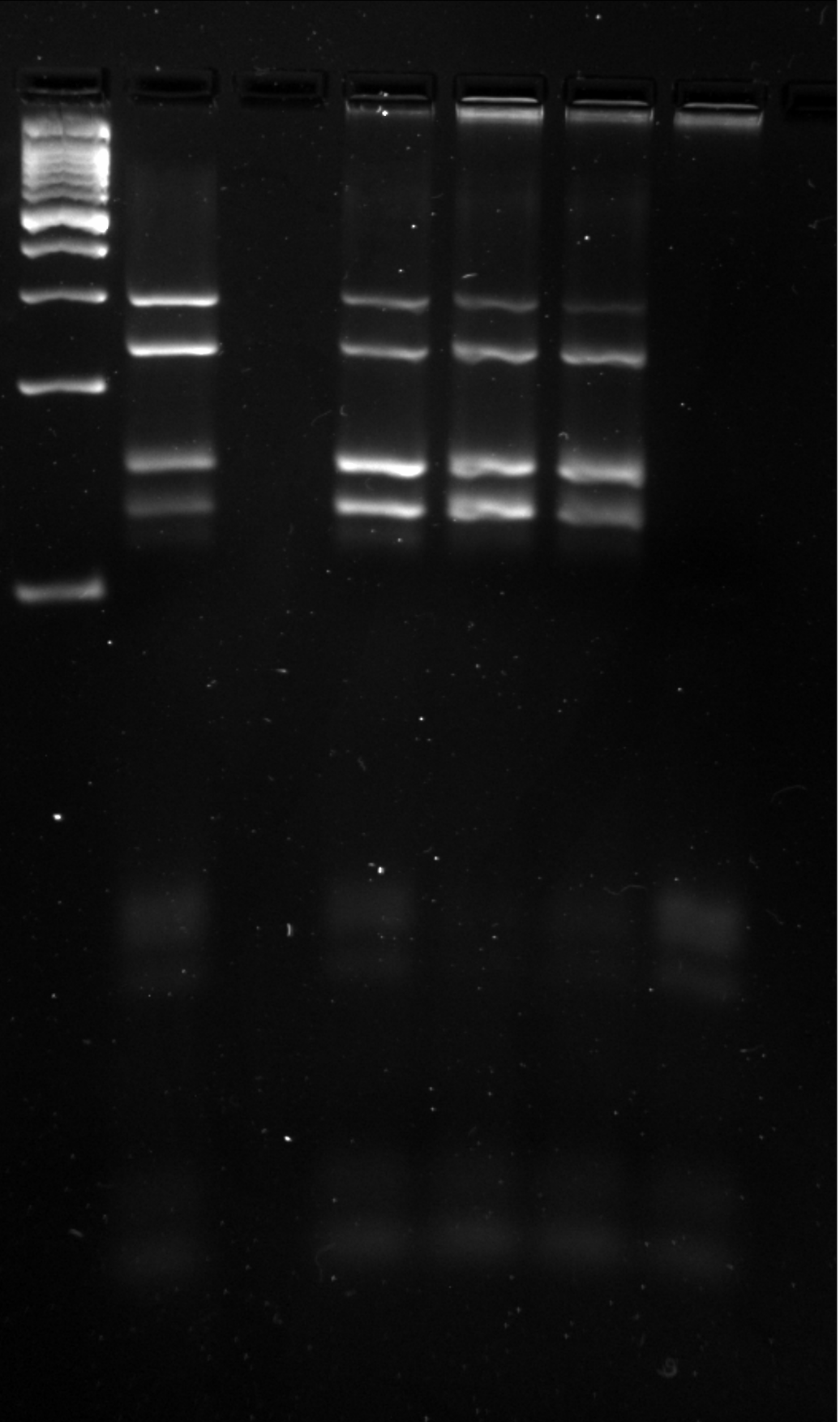

Supplement: Supplementary file 8 — Source Data [file 41467_2020_19394_MOESM8_ESM.zip › Source Data/Figure 6c, S6a, S9a_Gels/supp 5 chromosome gel.pdf]

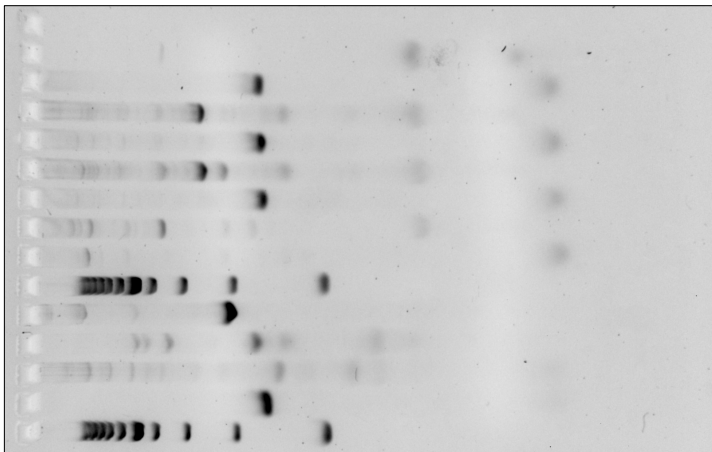

Supplement: Supplementary file 8 — Source Data [file 41467_2020_19394_MOESM8_ESM.zip › Source Data/Figure 6c, S6a, S9a_Gels/supp red green gel.pdf]
